# Randomized controlled trials on promoting self-care behaviors among informal caregivers of older patients: a systematic review and meta-analysis

**DOI:** 10.1186/s12877-023-04614-6

**Published:** 2024-01-23

**Authors:** Huanran Liu, Vivian W. Q. Lou, Shicheng Xu

**Affiliations:** 1 Department of Social Work & Social Administration, The University of Hong Kong, Pokfulam Road, Hong Kong SAR, China; 2 Sau Po Centre On Ageing, The University of Hong Kong, Hong Kong SAR, China

**Keywords:** Self-care, Self-management, Informal caregivers, Randomized controlled trial, Meta-analysis

## Abstract

**Background:**

Informal caregivers of older patients often neglect their self-care, despite the mental and physical health effects of caregiving. Randomized controlled trials (RCTs) on self-care interventions for informal caregivers are lacking, making it difficult to determine effective strategies. This systematic review explored the definition and categories of self-care RCTs for informal caregivers and a meta-analysis was conducted to determine the effectiveness of these interventions.

**Methods:**

Seven databases (Scopus, Web of Science, MEDLINE, PubMed, ProQuest, CINAHL, and Embase) were searched for articles in English reporting on self-care intervention outcomes for informal caregivers of patients aged 60 years or older. Standardized mean differences (SMD) with 95% confidence intervals (CI) were calculated using a random-effects model. Subgroup, sensitivity, and publication bias analyses were conducted.

**Results:**

Eighteen studies were included in the systematic review, of which fifteen studies were included in the meta-analysis. RCTs lacked a clear definition of self-care, mainly focused on promoting physical and mental health and individual capacity, and neglected disease prevention. The interventions focused on self-management for health and individual agency, with less attention on health literacy, decision-making capacity, self-monitoring for health status, and linkage to the health system. Meta-analysis results showed that RCTs had a small effect on reducing anxiety (SMD = -0.142, 95% CI [-0.302, 0.017], *p* = .081) but a significant effect on reducing depression (SMD = -0.214, 95% CI [-0.353, -0.076], *p* = .002). Country and type of caregiver significantly contributed to the effect of reducing caregivers’ depression in subgroup analysis.

**Conclusions:**

Studies on caregiver-centered self-care interventions are limited, resulting in a lack of a clear definition and comprehensive intervention. RCTs indicated a small effect on informal caregivers’ mental health, and interventions should consider both mental and physical health. More evidence is needed on the effectiveness of self-care interventions for informal caregivers’ anxiety and physical health.

**Supplementary Information:**

The online version contains supplementary material available at 10.1186/s12877-023-04614-6.

## Introduction

Informal caregivers are relatives, friends, or neighbors who provide ongoing assistance, typically unpaid, to someone with limitations in their physical, mental, or cognitive function [1, 2]. Their mental and physical health can be adversely affected by the role change and financial stress until the caregiving role ends [3]. As outlined by Pearlin’s stress process model, anxiety, depression, irascibility, and cognitive disturbance are important outcomes of caregivers’ mental health [4]. Self-care behaviors are an important contributor to health outcomes [5] that can reduce the effect of caregiver stress on general well-being [6]. According to the Embracing Carers International Global Survey, 42% of informal caregivers prioritized the health of the care recipients over their personal care in 2017, and this further increased to 89% in 2020 [7, 8]. More importantly, caregivers and care recipients share a reciprocal relationship [9]. Negative psychological emotions in caregivers have a negative impact on care recipients’ cognitive function [10] and dependence in activities of daily living (ADLs) [9]. In other words, if caregivers take good care of themselves, this will benefit both them and their care recipients. Therefore, it is important for caregivers to have more awareness of their health status and engage in health-promoting self-care behavior [6, 11], especially physical activity, stress management, social support, and support resources [12].

Self-care was first defined in 1983 by the World Health Organization (WHO) [13] and updated in 2013 as “the ability of individuals to promote and maintain health, prevent disease, and cope with illness and disability with or without the support of a healthcare provider” [14]. The concept of self-care has been developed and applied in the field of informal caregivers of older patients during recent decades. Self-care interventions are tools that support self-care [15], encompassing practices and approaches that intersect with health systems and health professionals [16]. Self-care interventions include but are not limited to self-management, self-testing, and self-awareness [5]. In 2022, WHO further classified these interventions into individual agency, health information-seeking, social and community support, personal health tracking, self-diagnosis of health conditions, self-management of health, health system, and financial aspects [15]. Self-care for caregivers is important, and some interventions have emerged to enable their self-care. However, current research on self-care interventions for caregivers shows limitations. First, self-care has often been defined as self-management, because they are often thought of as synonymous, making evidence relating to self-care interventions obscure and confusing [17]. Moreover, most interventions have focused on helping caregivers support the disease management of patients, rather than aiding the caregivers [18–20]. Although a scoping review of interventions for family caregiver self-care was completed, the results were not comprehensive because it only involved family caregivers of people with dementia [21]. People with dementia only account for 48% of all patients with informal caregivers, suggesting that self-care interventions for more than half of older patients’ caregivers remain unclear [22]. Given these limitations, this study systematically collected randomized controlled trials (RCTs) on promoting self-care behaviors among informal caregivers of older patients, including the definition of self-care and categories of self-care interventions in these studies. A meta-analysis followed to determine the effectiveness of these RCTs for informal caregivers’ self-care.

## Methods

This study was conducted based on the Preferred Reporting Items for Systematic Reviews and Meta-Analyses guidelines [23], as shown in Additional file 1. This review was registered on PROSPERO: CRD42023393329.

### Search strategy

A comprehensive search was conducted using seven electronic databases in the field of social science, gerontology, public health, medicine, and nursing: Scopus, Web of Science, MEDLINE, PubMed, ProQuest, CINAHL, and Embase. In November 2022, two independent reviewers searched titles, abstracts, and keywords for relevant journal articles published between January 1, 2000, and October 31, 2022, with language restricted to English. The general search strategies and queries are listed in Table 1. The detailed search strategy for each database is listed in Additional file 2. A social science librarian at the affiliated university enriched the search strategies.

**Table 1:** General search strategies and queries in the database

General queries	Boolean operators
“self-care” OR “self-management” OR “self-awareness” OR “self-testing” OR “self care” OR “self management” OR “self awareness” OR “self testing”	AND
“caregiver*” OR “carer*” OR “family caregiver*” OR “family carer*” OR “informal caregiver*” OR “informal carer*” OR “spouse caregiver*” OR “spouse carer*” OR “family member*” OR “non-professional care*” or “unpaid care*”	AND
“randomized controlled trial” OR “randomised controlled trial” OR “randomized and controlled trial” OR “randomised and controlled trial” OR “RCT” OR “pilot randomized controlled trial” OR “pilot randomised controlled trial” OR “pilot RCT” OR “randomized controlled pilot study” OR “randomised controlled pilot study”	AND NOT
“review” OR “systematic review” OR “meta analysis” OR meta-analysis OR “narrative review”	

### Selection criteria

Studies were included based on the following criteria: (a) patients aged 60 years old or older; (b) informal caregivers aged 18 years old or older; (c) RCT or pilot RCT; (d) included detailed intervention procedures and outcomes; (e) peer-reviewed; and (f) written and published in English. Studies were excluded if they were: (a) not caregiver-centered; (b) RCT protocol; (c) not published in a journal; or (d) not available as full text.

### Data extraction

The web-based literature review tool Covidence (http://www.covidence.org) was used to facilitate the systematic review process. After identifying all relevant articles and removing duplicates, two reviewers screened the titles, abstracts, and full-text articles together. Disagreements were addressed by the third researcher. The following components for each article were extracted by two reviewers together and stored and synthesized in Microsoft Excel: (a) author and publication year; (b) study country; (c) definition of self-care; (d) self-care category (based on the WHO Self-Care Framework); (e) study design (RCT or pilot RCT, single-blinded, double-blinded, or not blinded); (f) participants in the intervention group and control group; (g) care recipients’ diagnosis; (h) study intervention details (duration, intensity, type, and frequency); and (i) outcome.

### Assessment of risk of bias

The Cochrane risk of bias tool for RCTs was used to evaluate the methodological quality. It measures risks in seven domains: random sequence generation, allocation concealment, selective reporting, blinding of participants and personnel, blinding of outcome measurement, incomplete outcome data, and other bias [24]. Each domain is scored as “low” (low risk of bias), “high” (high risk of bias), or “unclear” (insufficient rationale or information for judgment). The result is determined by the number of “low” scores in each dimension, with less than or equal to two indicating high risk of bias, three to five indicating moderate risk of bias, and six or seven indicating low risk of bias. In this study, two reviewers independently assessed the risk of bias in each study, and disagreements were resolved by discussion with the third reviewer.

### Data synthesis and analysis

The meta-analysis was conducted using the “meta” package in R studio 2022.07.2. Because the outcomes were all continuous variables, this study used standardized mean differences (SMD) as a composite effect measure, along with 95% confidence intervals (CI). We extracted data on the number of participants, means, and standard deviations for the intervention and control groups after the intervention. Where standard deviations were not reported by the authors, they were calculated by the researchers using the formula (SD = SE × √n). For studies with multiple follow-ups, only the first outcome measurement after the intervention or follow-up was extracted for this study.

For each meta-analysis, statistical heterogeneity was assessed using the Cochran Q test and I^2^ statistic. Due to the various populations and criteria in different studies, this study used random-effects modeling to pool the results. Heterogeneity was indicated if the *p*-value was less than 0.05 and the I^2^ value was greater than 40% [25]. Subgroup analysis was also performed according to the country, intervention type, participants, type of patient, evaluation instruments, and outcome measure time. Heterogeneity tests assessed differences between studies using Q or I I^2^ statistics. If heterogeneity is significant (*p*-value < 0.05 or I^2^ > 50%), it indicates that effect sizes differ significantly across studies. Funnel plots and Egger’s test were used to assess publication bias. Sensitivity analyses were used to explore the robustness of the results, and pooled effect sizes were re-estimated after excluding studies at high risk of bias and compared with the meta-analysis results before exclusion. If no change in the results occurred, the conclusions obtained from this study were robust.

## Results

### Selection and characteristics of studies

**Fig. 1:**
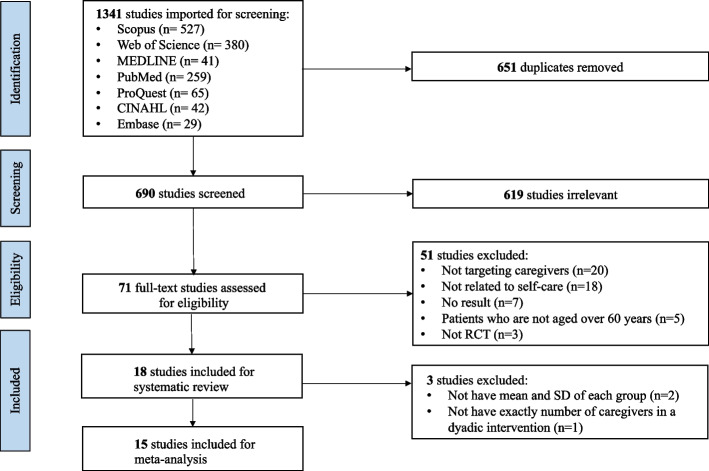
Flow diagram of search

Figure 1 summarizes the review process. The initial search yielded 1,341 articles from seven electronic databases, with 651 duplicates removed. After screening the titles and abstracts and reviewing full-text articles, 18 articles met the inclusion criteria for systematic review and 15 articles were included in the meta-analysis.

This systematic review included eighteen studies involving RCTs (details in Table 2). The sample sizes of these studies ranged from 26 to 642 individuals. These studies were published between 2006 and 2022. Ten studies were not blinded or did not describe blinding [26–35], five studies were single-blinded [36–40], and three studies were double-blinded [41–43]. Eight studies occurred in the United States [30–36, 41], one in Australia [38], three in the Netherlands [26, 37, 43], two in Hong Kong [40, 42], and one each in Japan [28], Singapore [27], Korea [39], and Germany [29]. Among these eighteen studies, seven studies involved caregivers and patients [27, 32, 34, 37, 38, 42, 43], whereas eleven studies involved only caregivers [26, 28–31, 33, 35, 36, 39–41]. In terms of the minimum age requirement for caregivers, the available literature presents varying findings. Nine studies established the minimum age for caregivers at 18 [26, 33, 35–38, 40–42], whereas four studies set the minimum age limit at either 21 or 40 years old [27, 30, 32, 39]. Additionally, five studies did not identify any specific age restrictions. It is noteworthy that most studies examined both male and female caregivers, with only two studies specifically focusing on the gender of caregivers: one study concentrating on female caregivers [30] and another on male caregivers [34]. Regarding the relationship between caregivers and patients, the majority of studies encompassed spouses, partners, relatives, and friends. However, two studies exclusively concentrated on spouses [30, 34], while one study specifically targeted adult children [42]. Eight studies focused on older patients with dementia and cognitive impairment [28–32, 37, 41, 42], five studies involved older patients with cancer [27, 33, 34, 36, 38], one study focused on patients who were depressed [26], one study involved patients with Parkinson’s disease [43], one study involved patients with chronic disease [40], one study targeted people under long-term care [39], and one study focused on patients with hematopoietic stem cell transplantation [35]. Thirteen studies used depression [27, 28, 30, 33, 34, 36–43] and six studies used anxiety [26, 33, 34, 36, 37, 40] as the mental health outcome measure; two studies used physical function as the outcome measure [30, 31]. The first outcome measurement time varied ranging from immediately postintervention to 6 months.

**Table 2:** Data extraction

Author (year), country	Definition of self-care	Self-care category	Study design	Participants	Characteristics of caregiver	Disease	Intervention	Outcome	First outcome measure
Dionne-Odom (2022), U.S. [36]	Promote and maintain mental health, improve individual capacity	Self-management of health, individual agency, and health information-seeking	Single-blinded, pilot RCT, IG (*n* = 31), CG (*n* = 32)	Caregivers	**Age**: Aged ≥ 18 years; **Gender**: Female and male; **Relationship with patients**: Spouse/partner; Parent; Other relative; Friend/other	Cancer	Six weekly 20- to 60-min telehealth coaching sessions plus monthly follow-up for 24 weeks, reviewing skills in stress management, self-care, getting help, staying organized, and future planning	Total distress; anxiety; depression; quality of life	2 months
Bijker (2017), Netherlands [26]	Promote and maintain mental health	Self-management of health	Not blinded, pilot RCT, IG (*n* = 41), CG (*n* = 39)	Caregivers	**Age**: Aged ≥ 18 years; **Gender**: Female and male; **Relationship with patients**: Parent; Child; Sibling; Other relative; Spouse/partner; Friend; Colleague/classmate; Other	Depressed	Eight nonsequential modules based on psychoeducation and CBT techniques; themes concerned information about depression, suicidality, communication and setting boundaries in caregiving, stress, burnout, and self-care	Primary outcome: User-friendliness; secondary outcomes: psychological distress, anxiety, subjective burden, quality of life, mastery	After intervention
Leow (2015), Singapore [27]	Promote and maintain mental health, improve individual capacity	Self-management of health, individual agency, social and community support	Not blinded, pilot RCT, IG (*n* = 38), CG (*n* = 42)	Caregivers and patients	**Age**: Aged ≥ 21 years; **Gender**: Female and male; **Relationship with patients**: Child; Spouse; Sibling; Parent; Niece; Daughter-in-law; Grandchild	Cancer	Psychoeducational intervention, the Caring for the Caregiver Programme, consisting of a 1-h face-to-face session, a video clip, two follow-up phone calls, and an invitation to an online social support group	Primary outcome: quality of life; secondary outcome: social support, stress and depression, self-efficacy in self-care, closeness with the patient, rewards, and knowledge	1 month
Fuju (2021), Japan [28]	Promote and maintain mental health	Self-management of health	Not blinded, RCT, IG (*n* = 13), CG (*n* = 13)	Caregivers	**Age**: NA; **Gender**: Female and male; **Relationship with patients**: Husband; Wife; Birth father; Birth mother; Father‐in‐law; Mother‐in‐law	Dementia	Positive diary in which participants wrote down three positive things that happened during the day, with reasons why they chose them, and compliment themselves at the end of each day; content not limited to caregiving	Primary outcome: depression; secondary outcome: quality of life, caregiver burden, positive cognitive appraisal, positive feelings	After intervention
Behrndt (2019), Germany [29]	Promote and maintain mental health, cope with illness and disability, improve individual capacity	Self-management of health, individual agency	Not blinded, RCT, IG (*n* = 205, dyadic), CG (n = 154, dyadic)	Caregivers	**Age**: NA; **Gender**: Female and male; **Relationship with patients**: Spouse; son/daughter (in-law); other	Cognitive impairment	Intervention group received counseling in three phone calls focused on stress reduction, development of self-management strategies, and how to deal with challenging behaviors	Primary outcome: subjective burden and depressiveness; secondary outcome: positive aspects of caregiving, health-related quality of life	6 months
Boots (2018), Netherlands [37]	Promote and maintain mental health, improve individual capacity	Self-management of health, individual agency	Single-blinded, RCT, IG (*n* = 41), CG (*n* = 40)	Caregivers and patients	**Age**: Aged > 18 years; **Gender**: Female and male; **Relationship with patients**: Spouse; other caregivers (e.g., children)	Dementia	8-week, blended care self-management Partner in Balance program, which combines face-to-face coaching with tailored web-based modules	Primary proximal outcome: self-efficacy; primary distal outcome: depression; secondary outcome: mastery, quality of life, and psychological complaints	After intervention
Terracciano (2020), U.S. [41]	Promote and maintain mental health, improve individual capacity	Self-management of health, individual agency, health information-seeking	Double-blinded, RCT, IG (*n* = 37), CG (*n* = 36)	Caregivers	**Age**: Aged ≥ 18 years; **Gender**: Female and male; **Relationship with patients**: Spouse; child; others	Dementia	Psychoeducational intervention that helps caregivers enhance self-care practices and manage emotional distress	Depressive symptoms, self-efficacy, self-rated health, and life satisfaction	After intervention
Connell (2009), U.S. [30]	Promote and maintain physical and mental health, improve individual capacity	Self-management of health, individual agency	Not blinded, RCT, IG (*n* = 86), CG (*n* = 71)	Caregivers	**Age**: Aged ≥ 40 years; **Gender**: Female; **Relationship with patients**: Wife	Dementia	Intervention group received 14 telephone calls from trained behavior-change counselors for 6 months	Self-rated physical health, count of chronic conditions, physical functioning, objective caregiving burden, exercise behavior, exercise self-efficacy, self-efficacy for self-care, and depressive symptoms	6 months
Heckel (2018), Australia [38]	Promote and maintain physical and mental health, improve individual capacity	Self-management of health, individual agency, social and community support, and individual financial transactions for health	Single-blinded, RCT, IG (*n* = 108, dyadic), CG (*n* = 108, dyadic)	Caregivers and patients	**Age**: Aged ≥ 18 years **Gender**: Female and male; **Relationship with patients**: Spouse/partner; other (e.g. parent, adult child, friend)	Cancer	Three outcalls from a nurse addressing six topics: psychological distress, health literacy, physical health, family support, financial burden, and practical difficulties (e.g., legal affairs)	Primary outcome: self-reported caregiver burden; secondary outcome: depressive symptoms, unmet needs, self-esteem, self-empowerment, and health literacy	1 month
Belle (2006), U.S. [32]	Promote and maintain mental health, cope with illness and disability, improve individual capacity	Self-management of health, individual agency, social and community support, and individual linkage to health system	Not blinded, RCT, IG (*n* = 323), CG (*n* = 319)	Caregivers and patients	**Age**: Aged ≥ 21 years **Gender**: Female and male; **Relationship with patients**: Spouse; child; sibling; other	Dementia	Twelve in-home and telephone sessions for 6 months	Primary outcome: quality of life; secondary outcomes: clinical depression and institutional placement of care recipient at 6 months	6 months
Nightingale (2022), U.S. [33]	Improve individual capacity	Self-management of health, individual agency, social and community support, and individual linkage to health system	Not blinded, pilot RCT, IG (*n* = 17), CG (*n* = 18)	Caregivers	**Age**: Aged ≥ 18 years; **Gender**: Female and male; **Relationship with patients**: Spouse or partner; other family member; friend	Cancer	6- to 7-week supported self-management intervention offering psychoeducation and stress management skills building.	Feasibility, acceptability, and self-efficacy for caregiving (total score and subscales)	After intervention
Lewis (2019), U.S. [34]	Improve individual capacity	Self-management of health, individual agency, social and community support	Not blinded, RCT, IG (*n* = 159), CG (*n* = 163)	Caregivers and patients	**Age**: NA; **Gender**: Male; **Relationship with patients**: Spouse (husband)	Cancer	Spouses in the experimental group received five 30- to 60-min intervention sessions at 2-week intervals by master’s-level patient educators	Depressed mood, anxiety, cancer-related marital communication, interpersonal support, and self‐care	3 months
El-Jawahri (2020), U.S. [35]	Promote and maintain physical and mental health, improve individual capacity	Self-management of health, individual agency	Not blinded, RCT, IG (*n* = 45), CG (*n* = 47)	Caregivers	**Age**: Aged ≥ 18 years; **Gender**: Female and male; **Relationship with patients**: Married; child; parent; sibling; divorced	Hematopoietic stem cell transplantation	Caregivers in intervention group met with a trained interventionist in person, via telephone, or via videoconferencing for 6 sessions starting before transplantation and continuing for up to 60 days	Quality of life, caregiving burden, psychological distress, self-efficacy, and coping	2 months
Au (2020), Hong Kong [42]	Promote and maintain mental health, improve individual capacity	Self-management of health, individual agency, social and community support	Double-blinded, RCT, IG (*n* = 37), CG (*n* = 35)	Caregivers and patients	**Age**: Aged ≥ 18 years; **Gender**: Female and male; **Relationship with patients**: Adult child	Dementia	8-week intervention: Connecting Through Caregiving with intergenerational perspective-taking reappraisals	Primary outcome: life satisfaction; secondary outcomes: depressive symptoms, burden, perspective-taking reappraisals	2 months
Han (2020), Korea [39]	Promote and maintain mental health, improve individual capacity	Self-management of health, individual agency	Single-blinded, RCT, IG (*n* = 498), CG (*n* = 471)	Caregivers	**Age**: Aged ≥ 20 years; **Gender**: Female and male; **Relationship with patients**: Spouse; son/daughter in law; other	Long-term care	Eight-week COMPASS program consisting of six individual in-home, three group support sessions, and two telephone sessions with a multicomponent intervention	Primary outcomes: depression, burden, and stress; secondary outcomes: self-efficacy, positive aspects of caregiving, social support, social activities, and health risk behaviors	2 months
Hou (2014), Hong Kong [40]	Promote and maintain mental health, improve individual capacity	Self-management of health, individual agency	Single-blinded, RCT, IG (*n* = 70), CG (*n* = 71)	Caregivers	**Age**: Aged ≥ 18 years; **Gender**: Female and male; **Relationship with patients**: Spouse; children; parents; relatives	Chronic conditions	Eight weekly 2-h sessions led by trained instructors; participants instructed to do CD-guided home practice for 30–45 min per day	Primary outcome: depressive symptoms; secondary outcome: anxiety symptoms, quality of life, self-efficacy, self-compassion, and mindfulness	After intervention
Elliott (2010), [31]	Promote and maintain mental health	Self-management of health, individual agency	Not blinded, RCT, IG (*n* = 257), CG (*n* = 238)	Caregiver	Age: NA; Gender: Female and male; Relationship with patients: Spouse; nonspouse	Dementia	Nine in-home and three telephone sessions over 6 months in the intervention group. Two brief “check-in” telephone calls during this 6-month period in the control group.	Primary outcome: health status; Secondary outcomes: burden; bother	6 months
A’Campo (2010) [43]	Promote and maintain mental health	Self-management of health, individual agency, social and community support	Double-blinded, RCT, IG (*n* = 26 caregivers), CG (*n* = 20 caregivers)	Caregivers and patients	Age: NA; Gender: Female and male; Relationship with patients: Partner; close relatives	Parkinson’s disease	Intervention group receive eight weekly standardized Patient Education Program Parkinson (PEPP). Control group receive usual neurological care	Psychosocial problems and need for help due to Parkinson’s disease; health-related quality of life (Hr-Qol); depression	After intervention

*IG* intervention group, *CG* control group

### Risk of bias assessment

**Fig. 2:**
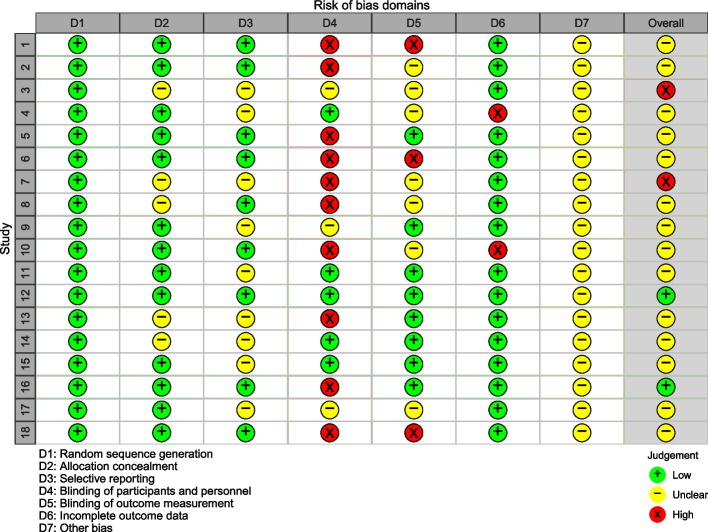
Risk of bias of 18 studies included in the systematic review. Studies: (1) Dionne-Odom, 2022 [36]; (2) Bijker, 2017 [26]; (3) Leow, 2015 [27]; (4) Fuju, 2021 [28]; (5) Boots, 2018 [37]; (6) Terracciano, 2020 [41]; (7) Connell, 2009 [30]; (8) Heckel, 2018 [38]; (9): Elliott, 2010 [31]; (10) Nightingale, 2022 [33]; (11) Lewis, 2019 [34]; (12) Au, 2020 [42]; (13) Han, 2020 [39]; (14) A’Campo, 2010 [43]; (15) Hou, 2014 [40]; (16) Behrndt, 2019 [29]; (17) Belle, 2006 [32]; (18) El-Jawahri, 2020 [35]

Figure 2 shows that two studies had low risk of bias [29, 42], three studies had high risk of bias [27, 30, 31], and the other thirdteen studies had moderate risk of bias. Most studies ensured randomization, allocation concealment, but ten studies have high risk in blinding of participants and personnel [26, 29, 30, 33, 35–39] and three studies have high risk in blinding of outcome measurement [35, 36, 41]. Nine studies [26, 29, 33, 35–38, 41, 42] had no selection reporting bias; the remainder could not be verified. Although most of the studies had reasonable attrition, only two studies had a remaining sample size of less than 30 participants, which we believe may have resulted in bias due to incomplete outcome data [28, 33]. We could not verify other risks of bias in these studies due to the lack of primary data, so other forms of bias in each study were unclear.

### Definition of self-care

The WHO’s definition of self-care was adopted in analyzing the data [14]. For studies that did not have a clear definition of self-care, we summarized the definition based on the objectives and intervention content. Table 2 shows all these studies regarded self-care as activities that promote and maintain physical or mental health status. Additionally, most studies considered individual capacity growth and empowerment, including self-efficacy [30, 33, 34, 37], communication skills [35, 39–41], health literacy [27, 35, 38, 40], decision-making ability [36, 38, 39, 41], and self-empowerment [29, 32] of caregivers. However, no studies focused on preventing certain diseases among caregivers, and only two studies focused on coping with illness and disability [29, 32], instead aiming to address care recipients’ behavior problems instead of caregivers.

Therefore, most studies defined self-care as activities or practices that promote and maintain physical and mental health and enhance individual capacity and empowerment, but very few studies addressed the prevention and management of diseases among caregivers.

### Category of self-care interventions

Regarding self-care interventions for self-carers and caregivers, this study classified these interventions into eight aspects. As shown in Table 2, all these studies fell in the “self-management of health” category, which includes self-care prevention that supports physical and mental health and well-being. Most studies also fell into the “individual agency” category, which encompasses promoting awareness of self-care, confidence and efficacy, self-care capacity, health and digital literacy, and sustained adoption of self-care practices and behaviors. Seven studies were classified in the “social and community support” category [27, 32–34, 38, 42, 43], which means these interventions can help caregivers get support from local networks, such as family, community, university, and the internet. Only two studies belonged to the “health information-seeking” category, related to acquiring health education for health-related decision-making [36, 41]. One study was classified as the “individual financial transactions for health” category, which involves financial support and practical difficulties (such as legal affairs) among caregivers [38]. No studies focused on personal health tracking (self-monitoring of health at home or in the community, data capture or documentation by self-care user or device), self-diagnosis of health conditions (self-testing and self-collection of samples for external testing), and individual linkage to the health system (identifying the location of health facilities and receiving feedback from health workers).

### Results of meta-analysis

Although these studies reported results from more than a dozen RCTs of caregiver self-care, such as burden, stress, self-efficacy, the only outcomes truly relevant to caregivers included mental health (depression, anxiety, irascibility, cognitive disturbance) and physical health, according to the stress process model presented by Pearlin in 1990 [4]. In these 15 studies, the mental health outcomes were anxiety and depression, whereas physical health outcomes were rare and not consistent with each other in conceptualization and operationalization. Subgroup analysis was also performed by country, intervention form, intervention duration, type of caregivers, participants, type of patients, evaluation instruments, and outcome measure time.

### Anxiety

**Fig. 3:**
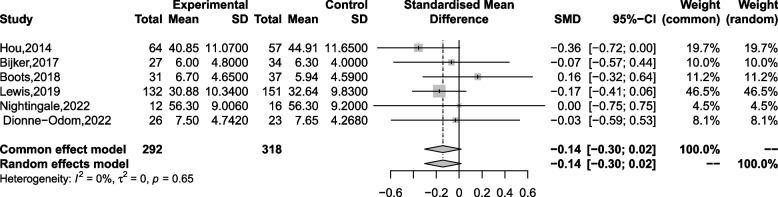
Forest plot of the effect of 6 studies on anxiety level

Six studies were included in a meta-analysis to evaluate the impact of current interventions on reducing the anxiety of caregivers. The result of the meta-analysis shows these interventions did not significantly affect the anxiety of caregivers (SMD = -0.142, 95% CI [-0.302, 0.017], *p* = 0.081; see Fig. 3) and had low heterogeneity (I^2^ = 0.0%, *p* = 0.646). No publication bias was found from the funnel plot (details in Additional file 3) and Egger’s test (*p* = 0.291). Considering the absence of studies of substandard quality within the selected pool of six studies, we conduct a leave-one-out approach for the sensitivity analysis and get the same results (SMD = -0.142, 95% CI [-0.302, 0.017], *p* = 0.081). However, none of the variables contributed significantly to the between-group variance in effect sizes, suggesting that these six studies did not differ by subgroup factors in reducing caregiver anxiety (details in Additional file 3).

### Depression

**Fig. 4:**
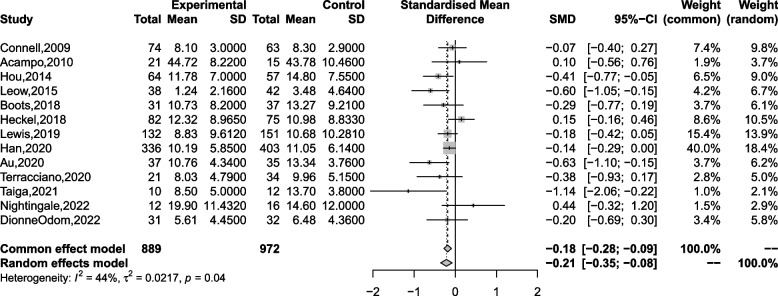
Forest plot of the effect of 13 studies on depression level

Thirteen studies were included in a meta-analysis to assess the effectiveness of these interventions in reducing depression in caregivers. Results show they significantly reduced depression among caregivers (SMD = -0.214, 95% CI [-0.353, -0.076], *p* = 0.002; see Fig. 4) and had moderate heterogeneity (I^2^ = 44.2%, *p* = 0.043). No publication bias was shown from the funnel plot (details in Additional file 4) and Egger’s test (*p* = 0.340). After excluding low-quality studies [27, 30], the results were stable after a sensitivity analysis of the remaining eleven studies (95% CI [-0.343, -0.053], *p* = 0.008).

As for subgroup analysis, we found that country and type of caregiver contributed significantly to the between-group variance in effect sizes (details in Table 3 and Additional file 4). Studies from Asia (Hong Kong, Singapore, and Japan) showed a stronger effect in reducing depression than other countries (*p* = 0.009). RCTs just involving family caregivers showed a stronger effect in reducing depression than all types of informal caregivers in these studies (*p* = 0.003).

**Table 3:** Subgroup analysis of depression

	K	SMD	95% CI	Q	*p*
*Country*					
United States	5	-0.145	[-0.311; 0.021]	16.840	.009
Netherlands	2	-0.154	[-0.542; 0.235]		
Hong Kong	2	-0.492	[-0.779; -0.205]		
Singapore	1	-0.603	[-1.052; -0.154]		
Australia	1	0.150	[-0.164; 0.463]		
Korean	1	-0.143	[-0.288; 0.002]		
Japan	1	-1.142	[-2.060; -0.225]		
*Intervention setting*					
Online	4	-0.114	[-0.366; 0.137]	1.000	.607
Face-to-face	6	-0.276	[-0.586; 0.034]		
Combination	3	-0.284	[-0.564; -0.005]		
*Duration*					
< 1 month	3	-0.502	[-0.973; -0.032]	5.890	.053
1–3 months	8	-0.222	[-0.370; -0.075]		
> 3 months	2	0.049	[-0.181; 0.278]		
*Caregiver type*					
Family	10	-0.277	[-0.408; -0.146]	9.020	.003
Informal	3	0.177	[-0.089; 0.443]		
*Participant type*					
Caregivers	7	-0.178	[-0.294; -0.062]	0.150	.700
Caregivers and patients	6	-0.234	[-0.493; 0.026]		
*Patient type*					
Dementia	5	-0.380	[-0.666; -0.094]	2.570	.463
Parkinson’s	1	0.100	[-0.563; 0.763]		
Cancer	5	-0.119	[-0.406; 0.167]		
No specific disease, in long-term care	2	-0.225	[-0.469; 0.018]		
*Measures*					
CES-D	10	-0.196	[-0.343; -0.050]	3.800	.284
SDS	1	0.100	[-0.563; 0.763]		
DASS	1	-0.603	[-1.052; -0.154]		
HADS	1	-0.195	[-0.690; 0.300]		
*Measurement time*					
Post-intervention	8	-0.275	[-0.467; -0.083]	1.180	.881
1 month	2	-0.209	[-0.946; 0.528]		
2 months	1	-0.195	[-0.690; 0.300]		
3 months	1	-0.185	[-0.419; 0.049]		
> 3 months	1	-0.067	[-0.403; 0.269]		

*CES-D* Center for Epidemiologic Studies Depression Scale, *SDS* Self-Rated Depression Scale, *DASS* Depression and Anxiety Stress Scales, *HADS* Hospital Anxiety and Depression Scale

### Physical function

Two studies focused on physical health, one focused on the improvement of self-rated physical health status, and another explored the exercise behavior of participants. Considering the limited studies and inconsistent variables, we could not conduct a meta-analysis of caregivers’ physical health. But RCT results suggest that interventions for caregivers can increase their exercise behavior and improve their self-rated physical health [31].

## Discussion

To our knowledge, this is the first systematic review of RCTs to promote self-care behavior among informal caregivers of older patients, with effectiveness examined by meta-analysis. From this review, we found that most existing RCTs conflated caregiver self-care with the self-management of patients, which is consistent with previous studies [17]. Meanwhile, some studies included self-care intervention as one of many subdomains, which made it difficult to affirm its true effectiveness [44]. As a result, it is clear that caregivers’ self-care has been overlooked and understudied.

Few RCTs have clearly defined self-care. Sakuma illustrated two types of self-care—direct provision of self-care technologies and indirect help with involvement in patient care—but this is not caregiver-centered self-care [45]. Although the WHO proposed a definition of self-care 40 years ago [13], no RCTs on caregivers’ self-care have used this definition. Based on this framework, our study defined the concept of self-care for each article and found that most studies focused on maintaining the physical and mental health of caregivers and promoting caregiver capacity related to caregiving, but they all neglected the prevention of future illnesses among caregivers, although caregiving often has a negative impact on both the physical and mental health of informal caregivers for older adults [2].

After categorizing these RCTs, we found that most focused on self-management for health and individual agency, but these studies only emphasized the importance of personal care, instead of teaching caregivers how to self-monitor their health status in daily life. Besides, few studies have paid attention to the importance of health-related decision-making, a critical issue because older patients and their caregivers often report low levels of self-perceived health literacy and low confidence in the information available to assess health-related decision-making [46]. Additionally, caregivers need social and local community support to avoid social isolation, cope with financial affairs, and engage in personal health care activities [12]. Therefore, future interventions on self-care for older patients’ caregivers should focus on building capacity for decision-making and establishing links between individuals and the health system.

Regarding the outcomes of these RCTs, we found that they mostly focused on caregivers’ mental health, with physical health rarely appearing as the outcome. This result is consistent with another systematic review on family caregivers’ health status [47]. Although the physical effects of caregiving are generally less intensive and unnoticeable than the psychological effects [48], physical health is as important as psychological health and often affected by mental health [49]. In addition, mental health outcomes were mainly depression and anxiety, with no mention of irritability and cognitive impairment as mentioned in Pearlin’s stress process model [4]. Hence, this study suggests practitioners involved in caregiver self-care could focus on improvements in caregivers’ physical health and cognitive function.

We noticed that very few studies measure caregivers’ self-care behavior, one study measures caregivers' confidence in helping themselves deal with the demands and challenges of the patient’s disease instead of their own health self-care [34], but this is not caregiver-centered self-care. Only two studies measure the self-efficacy in taking care of themselves [30] and obtaining respite and controlling upsetting thoughts about the caregiving situation [27] but do not focus on the improvement of self-care ability. In other words, the measurement of caregivers’ self-care in research has been notably lacking. Moreover, the existing studies that have examined caregivers’ self-care abilty have predominantly concentrated on subjective assessments of self-care efficacy, rather than objective evaluations of actual self-care behaviors. Consequently, it is imperative for future research endeavors to place emphasis on directly measuring both the competence and efficacy of caregivers’ self-care. This comprehensive approach would enable the development of interventions that genuinely prioritize the unique needs and preferences of caregivers. By adopting such an approach, caregiver-centered interventions can be truly aligned with the well-being and specific requirements of the caregivers themselves.

The heterogeneity of caregivers’ anxiety was not analyzed in the subgroup due to limited studies. Country and type of caregiver proved to be reasons for heterogeneity in these studies regarding informal caregivers’ depression. In studies from Asia—including Japan, Singapore, and Hong Kong—the intervention was more effective in reducing depression among caregivers compared to the control group. This may be because of the importance of filial piety in most Asian countries, such that filial piety can protect informal caregivers from depression by altering appraisals of the caregiver role [50–52]. The type of caregiver was another reason for the heterogeneity of RCTs in reducing depression. These interventions were more effective for family caregivers than informal caregivers. Compared with informal caregivers, family caregivers often have a stronger emotional bond with care recipients, which might motivate them to take better care of themselves to ensure better care to older patients.

As for the intervention format, our subgroup results also confirm a combination of face-to-face and online intervention was more beneficial for caregivers compared to the control group [21], with only face-to-face or only online interventions (such as telephone-based interventions) not significantly different between control and intervention groups. Currently, telephone-based interventions for caregiver self-care are becoming increasingly popular with researchers, but more evidence is needed to verify their effectiveness. RCTs conducted within 3 months indicated the interventions were more effective in reducing informal caregivers’ depressive symptoms than in control groups, which suggests that future RCTs need to pay more attention to the durability of intervention effects with longer follow-up sessions [53].

We also examined the participants, types of patients, and evaluation instruments during subgroup analysis. However, improvements in caregivers’ mental health and physical health did not differ depending on these factors. Therefore, the inclusion of patients in the intervention is a possible direction to pursue to improve the self-care ability of both patients and caregivers. Previous studies have shown the benefits of dyadic intervention for patients [54], but the effects of dyadic interventions on the mental and physical health of caregivers need to be further explored. Because we focused on caregivers’ self-care outcomes, the effectiveness of these RCTs did not differ by the patients’ illness. But considering the limited studies in this meta-analysis, this finding still needs to be validated by more RCTs and meta-analyses.

Measurements of physical health in the included studies were not well established or widely used in caregiver self-care interventions. Caregivers’ physical health was not used as an outcome of most interventions, but rather as basic information about participants at baseline. Measurements of physical health were less consistent in two studies, which only used a self-reported questionnaire testing exercise condition and self-rated improvement in physical health, respectively [30, 31]. Although improvements in physical health are not achievable in the short term, we still recommend that physical health be valued in these interventions and assessed as an outcome.

Although quality of life is related to both physical and mental health dimensions and can also reflect the effectiveness of self-care interventions, it is a multidimensional concept that can be either a health-based approach, determined by the severity of the illness and the quality of care, or a person-centered approach, which is determined by the individual’s experience, subjective interpretations of health and illness and personal knowledge [55]. This heterogeneity poses challenges in conducting a reliable meta-analysis. Some studies adopted health-related approaches, focusing on the impact of caregiving on caregivers’ health-related quality of life (Hr-Qol) [26, 43]. Others employed person-centered approaches, capturing subjective aspects of quality of life, such as caregivers’ perceptions of their position in life and their overall well-being [27, 28, 33, 36, 37]. There was also one study that encompassed both physical and mental health dimensions in its measurement of quality of life [40]. While we acknowledge that quality of life is an important outcome measure in the context of self-care interventions for caregivers, the heterogeneity and conceptual challenges associated with its measurement within the included studies warranted caution in its inclusion in our meta-analysis. To ensure the reliability and validity of our meta-analysis results, we chose to focus on outcome measures that exhibited greater consistency and comparability across studies, such as depressive symptoms and anxiety.

This study has several limitations. To begin, the generalizability of our results may be limited because we only included studies in English. Additionally, we did not search for each subdimension under the WHO’s self-care framework, which prevented us from examining existing interventions in greater detail. Future reviews should focus on self-care interventions for informal caregivers based on each subdimension. Because different databases have different starting points of data collection, we restricted our time frame to 2000 onward and thus, records before 2000 were not explored. Finally, the effectiveness of RCTs on caregiver anxiety and physical function were not verified in our meta-analysis, which may be due to the limited number of included studies.

## Conclusion

Self-care for caregivers of older patients is an emerging topic. Based on the framework of self-care from the WHO, this study suggests that informal caregiver self-care should focus on the maintenance of both physical health and mental well-being and promote individual capacity and illness prevention. RCTs have mainly focused on self-management for health and individual agency and neglected education to improve health literacy, decision-making capacity, self-monitoring of health status, and access to resources from the community and health system.

The results of the meta-analysis indicated small associations between informal caregivers’ self-care interventions with their mental health. This study suggests that in addition to caregivers’ mental health, we should also focus on improving their physical health. The results of our sensitivity analysis show that our results are robust and stable, but due to the limited studies in the meta-analysis, the results of this subgroup analysis can only provide us with preliminary knowledge. More evidence from RCTs is needed on the effectiveness of informal caregivers’ self-care.

### Supplementary Information

**Additional file 1.** PRISMA 2020 Checklist.

**Additional file 2.** Search strategies in different databases.

**Additional file 3.** Meta-analysis for anxiety.

**Additional file 4.** Meta-analysis of depression.

## Declarations

### Availability of data and materials

The datasets used and/or analysed during the current study are available from the corresponding author on reasonable request.
