# Supplementary material for: Randomized controlled trials on promoting self-care behaviors among informal caregivers of older patients: a systematic review and meta-analysis
Source: BMC Geriatr. 2024 Jan 23;24:86. doi: 10.1186/s12877-023-04614-6 (PMC10804633; doi:10.1186/s12877-023-04614-6)
Supplement: Supplementary file 2 — Additional file 2. Search strategies in different databases. [file 12877_2023_4614_MOESM2_ESM.docx]

**Additional file 2. Search strategies in different databases**

**Scopus (Filters: title, abstract, keywords, with peer reviewed, English, article, 527 records found)**

TITLE-ABS-KEY ( ( self-care  OR  self-management  OR  self-awareness  OR  self-testing  OR  "self care"  OR  "self management"  OR  "self awareness"  OR  "self testing" )  AND  ( caregiver*  OR  carer*  OR  "family caregiver*"  OR  "family carer*"  OR  "informal caregiver*"  OR  "informal carer*"  OR  "spouse caregiver*"  OR  "spouse carer*"  OR  "family member*"  OR  "non-professional care*"  OR  "unpaid care*" )  AND  ( "randomi?ed controlled trial"  OR  "randomi?ed and controlled trial"  OR  "RCT"  OR  "pilot randomi?ed controlled trial"  OR  "pilot RCT"  OR  "randomi?ed controlled pilot study" )  AND NOT  ( "review"  OR  "systematic review"  OR  "meta analysis"  OR  meta-analysis  OR  "narrative review" ) )  AND  ( LIMIT-TO ( PUBSTAGE ,  "final" ) )  AND  ( LIMIT-TO ( DOCTYPE ,  "ar" ) )  AND  ( LIMIT-TO ( LANGUAGE ,  "English" ) )  AND  ( LIMIT-TO ( SRCTYPE ,  "j" ) )

**Web of science (Filters: topic(title, abstract, keywords, 380 records found)**

(((TS=(self-care OR self-management OR self-awareness OR self-testing OR “self care” OR “self management" OR "self awareness" OR "self testing")) AND TS=(caregiver* OR carer* OR "family caregiver*" OR "family carer*" OR  “informal caregiver*" OR "informal carer*" OR "spouse caregiver*" OR "spouse carer*" OR "family member*" OR "non-professional care*" or “unpaid care*")) AND TS=("randomi?ed controlled trial" OR "randomi?ed and controlled trial” OR “RCT” OR "pilot randomi?ed controlled trial" OR "pilot RCT" OR “randomi?ed controlled pilot study")) NOT TS=("review" OR "systematic review" OR "meta analysis" OR meta-analysis OR "narrative review”)

**MEDLINE (via Ovid, Filters: keywords, full-text, article, MEDLINE, n = 41)**

1. self-care.mp or exp Self Care/
2. self-management.mp or exp Self-Management/
3. self-awareness.mp or exp self-awareness/
4. self-testing.mp or exp self-testing/
5. "self care".mp. or exp Self Care/
6. "self management".mp. or exp Self-Management/
7. "self awareness".mp. or exp "self awareness"/
8. "self testing".mp. or exp "self testing"/
9. 1 or 2 or 3 or 4 or 5 or 6 or 7 or 8
10. caregiver*.mp.
11. carer*.mp.
12. "family caregiver*".mp.
13. "family carer*".mp.
14. "informal caregiver*".mp.
15. "informal carer*".mp.
16. "spouse caregiver*".mp.
17. "spouse carer*".mp.
18. "family member*".mp.
19. "non-professional care*".mp.
20. "unpaid care*".mp.
21. 10 or 11 or 12 or 13 or 14 or 15 or 16 or 17 or 18 or 19 or 20
22. "randomized controlled trial".mp.
23. "randomised controlled trial".mp.
24. "[randomized and controlled trial](https://idp.springer.com/authorize/casa?redirect_uri=https://link.springer.com/article/10.1007/s11325-009-0311-1&casa_token=vw4aHqOPiXQAAAAA:IVzqY82TCwc0gGn_zYWoGoVH2Lko6uTRSBiLa1WSzPhzXgAODSMasXAdMiUPNsg6PAnAPFp1RImmASTgJYQ)".mp.
25. "[randomised and controlled trial](https://idp.springer.com/authorize/casa?redirect_uri=https://link.springer.com/article/10.1007/s11325-009-0311-1&casa_token=vw4aHqOPiXQAAAAA:IVzqY82TCwc0gGn_zYWoGoVH2Lko6uTRSBiLa1WSzPhzXgAODSMasXAdMiUPNsg6PAnAPFp1RImmASTgJYQ)".mp.
26. "RCT".mp.
27. "pilot randomized controlled trial".mp.
28. "pilot randomised controlled trial".mp.
29. "pilot RCT".mp.
30. "[randomized controlled pilot study](https://link.springer.com/article/10.1186/1471-2431-11-97)".mp.
31. "randomised controlled pilot study".mp.
32. 22 or 23 or 24 or 25 or 26 or 27 or 28 or 29 or 30 or 31
33. 9 and 21 and 32
34. limit 33 to (english language and yr="2000 -Current" and english and medline)

**PubMed (Filters: title/ abstract, English, full-text, 259 records found)**

(((self-care[Title/Abstract] OR self-management[Title/Abstract] OR self-awareness[Title/Abstract] OR self-testing[Title/Abstract] OR "self care"[Title/Abstract] OR "self management"[Title/Abstract] OR "self awareness"[Title/Abstract] OR "self testing"[Title/Abstract]) AND (caregiver*[Title/Abstract] OR carer*[Title/Abstract] OR "family caregiver*"[Title/Abstract] OR "family carer*"[Title/Abstract] OR "informal caregiver*"[Title/Abstract] OR "informal carer*"[Title/Abstract] OR "spouse caregiver*"[Title/Abstract] OR "spouse carer*"[Title/Abstract] OR "family member*"[Title/Abstract] OR "non-professional care*"[Title/Abstract] OR "unpaid care*"[Title/Abstract])) AND ("randomized controlled trial"[Title/Abstract] OR "randomised controlled trial"[Title/Abstract] OR "randomized and controlled trial"[Title/Abstract] OR "randomised and controlled trial"[Title/Abstract] OR "RCT"[Title/Abstract] OR "pilot randomized controlled trial"[Title/Abstract] OR "pilot randomised controlled trial"[Title/Abstract] OR "pilot RCT"[Title/Abstract] OR "randomized controlled pilot study" [Title/Abstract] OR "randomised controlled pilot study"[Title/Abstract] )) NOT ("review"[Title/Abstract] OR "systematic review"[Title/Abstract] OR "meta analysis"[Title/Abstract] OR meta-analysis[Title/Abstract] OR "narrative review"[Title/Abstract])

**ProQuest: (Filters: all abstract and summary text and title with full text and peer review, English, article, 65 records found)**

summary(self-care OR self-management OR self-awareness OR self-testing OR "self care" OR "self management" OR "self awareness" OR "self testing") AND summary(caregiver* OR carer* OR "family caregiver*" OR "family carer*" OR "informal caregiver*" OR "informal carer*" OR "spouse caregiver*" OR "spouse carer*" OR "family member*" OR "non-professional care*" OR "unpaid care*") AND summary("randomized controlled trial" OR "randomised controlled trial" OR "[randomized and controlled trial](https://idp.springer.com/authorize/casa?redirect_uri=https://link.springer.com/article/10.1007/s11325-009-0311-1&casa_token=vw4aHqOPiXQAAAAA:IVzqY82TCwc0gGn_zYWoGoVH2Lko6uTRSBiLa1WSzPhzXgAODSMasXAdMiUPNsg6PAnAPFp1RImmASTgJYQ)" OR "[randomised and controlled trial](https://idp.springer.com/authorize/casa?redirect_uri=https://link.springer.com/article/10.1007/s11325-009-0311-1&casa_token=vw4aHqOPiXQAAAAA:IVzqY82TCwc0gGn_zYWoGoVH2Lko6uTRSBiLa1WSzPhzXgAODSMasXAdMiUPNsg6PAnAPFp1RImmASTgJYQ)" OR “RCT” OR "pilot randomized controlled trial" OR "pilot randomised controlled trial"  OR "pilot RCT"  OR "[randomized controlled pilot study](https://link.springer.com/article/10.1186/1471-2431-11-97)" OR "randomised controlled pilot study") NOT summary("review" OR "systematic review" OR "meta analysis" OR meta-analysis OR "narrative review")

**CINAHL (via EBSCO Host, filters: Abstract&Title, 42 records found)**

(self-care OR self-management OR self-awareness OR self-testing OR "self care" OR "self management" OR "self awareness" OR "self testing") AND
(caregiver* OR carer* OR "family caregiver*" OR "family carer*" OR  "informal caregiver*" OR "informal carer*" OR "spouse caregiver*" OR "spouse carer*" OR "family member*" OR "non-professional care*" or "unpaid care*") AND

("randomized controlled trial" OR "randomised controlled trial" OR "[randomized and controlled trial](https://idp.springer.com/authorize/casa?redirect_uri=https://link.springer.com/article/10.1007/s11325-009-0311-1&casa_token=vw4aHqOPiXQAAAAA:IVzqY82TCwc0gGn_zYWoGoVH2Lko6uTRSBiLa1WSzPhzXgAODSMasXAdMiUPNsg6PAnAPFp1RImmASTgJYQ)" OR "[randomised and controlled trial](https://idp.springer.com/authorize/casa?redirect_uri=https://link.springer.com/article/10.1007/s11325-009-0311-1&casa_token=vw4aHqOPiXQAAAAA:IVzqY82TCwc0gGn_zYWoGoVH2Lko6uTRSBiLa1WSzPhzXgAODSMasXAdMiUPNsg6PAnAPFp1RImmASTgJYQ)" OR “RCT” OR "pilot randomized controlled trial" OR "pilot randomised controlled trial"  OR "pilot RCT"  OR "[randomized controlled pilot study](https://link.springer.com/article/10.1186/1471-2431-11-97)" OR "randomised controlled pilot study") NOT

("review" OR "systematic review" OR "meta analysis" OR meta-analysis OR "narrative review")

**Embase (via Ovid, Filters: keywords, full-text, article, EMBASE and EMBASE status, 29 records found)**

1. self-care.mp or exp Self Care/
2. self-management.mp or exp Self-Management/
3. self-awareness.mp or exp self-awareness/
4. self-testing.mp or exp self-testing/
5. "self care".mp. or exp Self Care/
6. "self management".mp. or exp Self-Management/
7. "self awareness".mp. or exp "self awareness"/
8. "self testing".mp. or exp "self testing"/
9. 1 or 2 or 3 or 4 or 5 or 6 or 7 or 8
10. caregiver*.mp.
11. carer*.mp.
12. "family caregiver*".mp.
13. "family carer*".mp.
14. "informal caregiver*".mp.
15. "informal carer*".mp.
16. "spouse caregiver*".mp.
17. "spouse carer*".mp.
18. "family member*".mp.
19. "non-professional care*".mp.
20. "unpaid care*".mp.
21. 10 or 11 or 12 or 13 or 14 or 15 or 16 or 17 or 18 or 19 or 20
22. "randomized controlled trial".mp.
23. "randomised controlled trial".mp.
24. "[randomized and controlled trial](https://idp.springer.com/authorize/casa?redirect_uri=https://link.springer.com/article/10.1007/s11325-009-0311-1&casa_token=vw4aHqOPiXQAAAAA:IVzqY82TCwc0gGn_zYWoGoVH2Lko6uTRSBiLa1WSzPhzXgAODSMasXAdMiUPNsg6PAnAPFp1RImmASTgJYQ)".mp.
25. "[randomised and controlled trial](https://idp.springer.com/authorize/casa?redirect_uri=https://link.springer.com/article/10.1007/s11325-009-0311-1&casa_token=vw4aHqOPiXQAAAAA:IVzqY82TCwc0gGn_zYWoGoVH2Lko6uTRSBiLa1WSzPhzXgAODSMasXAdMiUPNsg6PAnAPFp1RImmASTgJYQ)".mp.
26. "RCT".mp.
27. "pilot randomized controlled trial".mp.
28. "pilot randomised controlled trial".mp.
29. "pilot RCT".mp.
30. "[randomized controlled pilot study](https://link.springer.com/article/10.1186/1471-2431-11-97)".mp.
31. "randomised controlled pilot study".mp.A
32. 22 or 23 or 24 or 25 or 26 or 27 or 28 or 29 or 30 or 31
33. 9 and 21 and 32
34. limit 33 to (embase and english and article and journal)
35. limit 34 to yr="2000 -Current"
36. limit 35 to "remove medline records"
