# Supplementary material for: Randomized controlled trials on promoting self-care behaviors among informal caregivers of older patients: a systematic review and meta-analysis
Source: BMC Geriatr. 2024 Jan 23;24:86. doi: 10.1186/s12877-023-04614-6 (PMC10804633; doi:10.1186/s12877-023-04614-6)

Additional file 3. Meta-analysis for anxiety

Forest plot


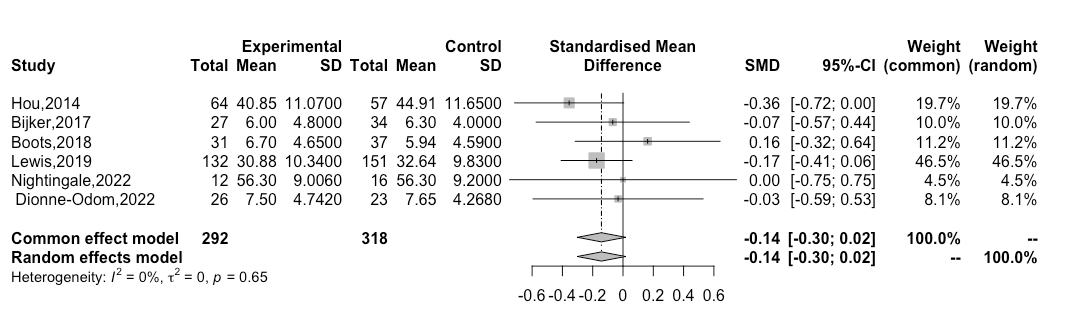


Funnel plot


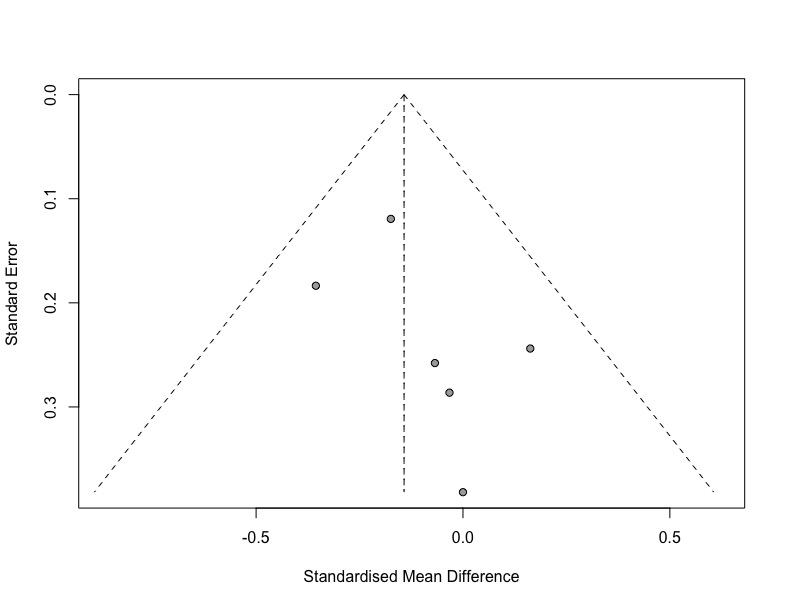


Subgroup analysis of anxiety

Table 1 Subgroup analysis of anxiety

|  | Coding | K (number) | SMD | 95% CI | Q (between groups) | P-value (between groups) |
| --- | --- | --- | --- | --- | --- | --- |
| Country | Hong Kong, SAR, China | 1 | -0.356 | [-0.715; 0.004] | 2.570 | 0.276 |
|  | The Netherlands | 2 | 0.054 | [-0.293; 0.401] |  |  |
|  | USA | 3 | -0.948 | [-0.349; 0.066] |  |  |
| Intervention | Online intervention | 3 | -0.210 | [-0.470; 0.050] | 1.850 | 0.397 |
| forms | Face-to-face intervention | 2 | -0.159 | [-0.382; 0.065] |  |  |
|  | Combination of online and face-to-face intervention | 1 | 0.163 | [-0.315; 0.641] |  |  |
| Intervention | Within one month or less | 1 | -0.174 | [-0.408; 0.060] | 0.160 | 0.686 |
| duration | One to three months | 5 | -0.106 | [-0.340; 0.128] |  |  |
| Type of | Family caregiver | 4 | -0.159 | [-0.331; 0.014] | 0.240 | 0.628 |
| caregiver | Informal caregiver | 2 | -0.047 | [-0.465; 0.372] |  |  |
| Participants | Caregiver | 4 | -0.188 | [-0.433; 0.058] | 0.330 | 0.564 |
|  | Caregiver& patients | 2 | -0.073 | [-0.376; 0.230] |  |  |
| Type of  patients | No specific disease in long-term care | 1 | -0.356 | [-0.715; 0.004] | 3.000 | 0.392 |
|  | Depressed | 1 | -0.068 | [-0.573; 0.438] |  |  |
|  | Dementia | 1 | 0.163 | [-0.315; 0.641] |  |  |
|  | Cancer | 3 | -0.142 | [-0.349; 0.066] |  |  |
| Evaluation | STAI | 2 | -0.228 | [-0.424; -0.032] | 2.400 | 0.494 |
| instruments | GAD-7 | 1 | -0.068 | [-0.573; 0.438] |  |  |
|  | HADS | 2 | 0.081 | [-0.283; 0.444] |  |  |
|  | EDA | 1 | 0.000 | [-0.749; 0.749] |  |  |
| Measure | Post-intervention | 4 | -0.112 | [-0.387; 0.164] | 0.260 | 0.877 |
| time | Follow 2 months | 1 | -0.033 | [-0.594; 0.529] |  |  |
|  | Follow 3 months | 1 | -0.174 | [-0.408; 0.060] |  |  |

P<0.1*, P<0.05**, P<0.01***

STAI, State-Trait Anxiety Inventory; GAD-7, Generalized anxiety disorder scale; HADS, Hospital Anxiety and Depression Scale (HADS); EDA, Emotional Distress Anxiety Short Form- 8a.

Subgroup 1- country


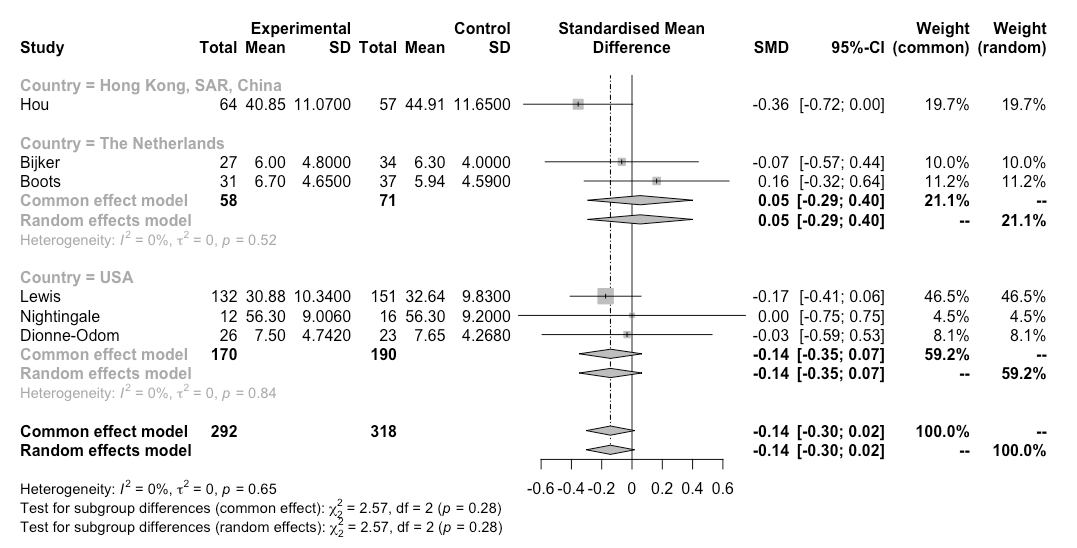


Subgroup 2 - intervention forms


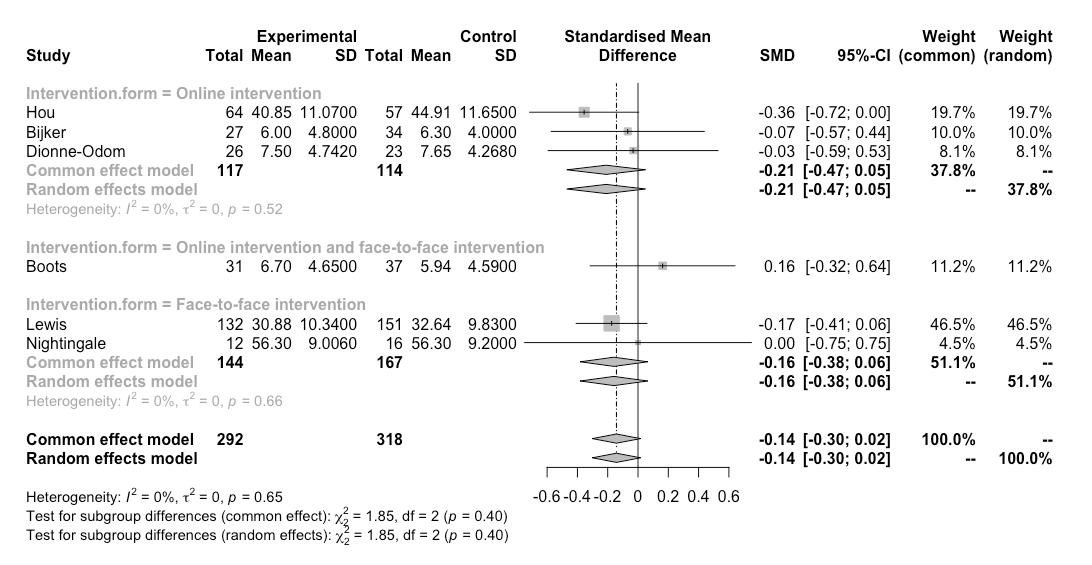


Subgroup 3-intervention duration


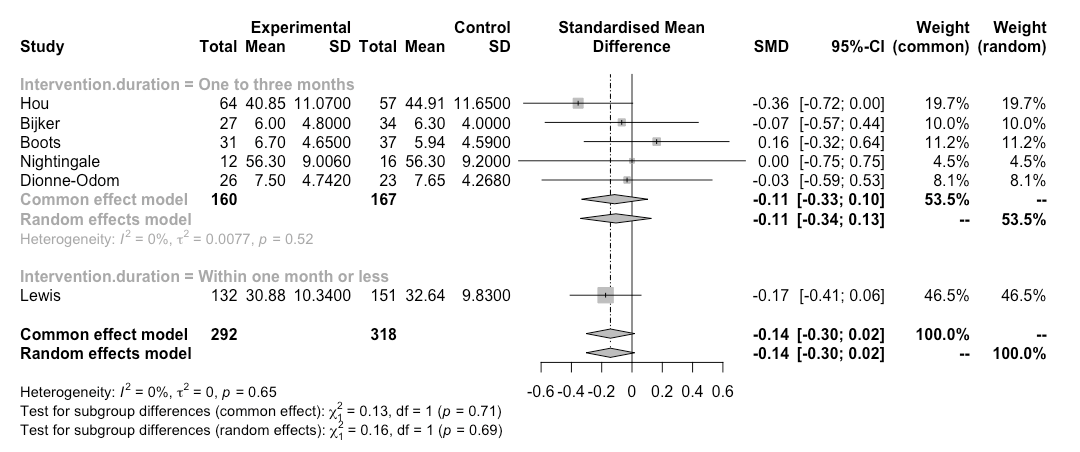


Subgroup 4- type of caregiver


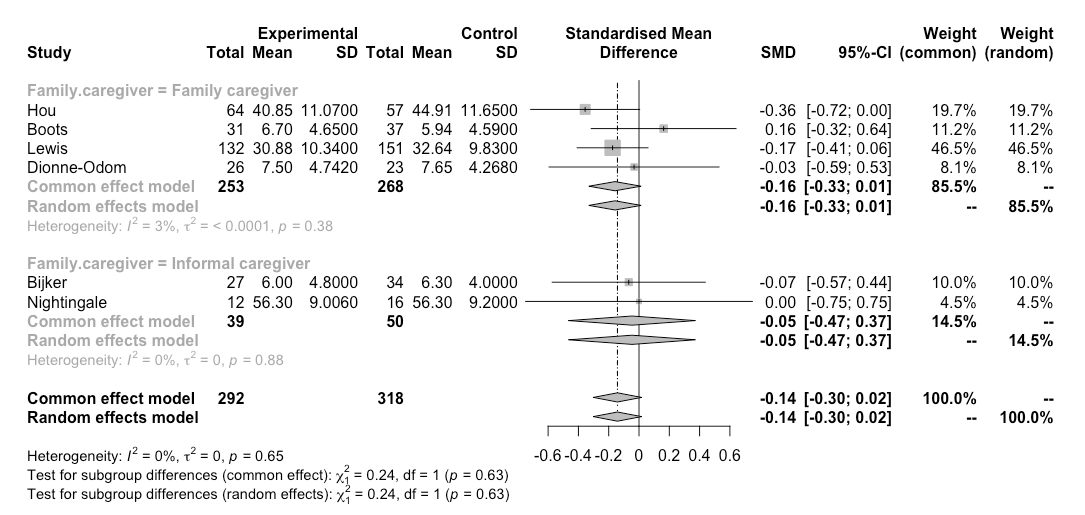


Subgroup 5- participants


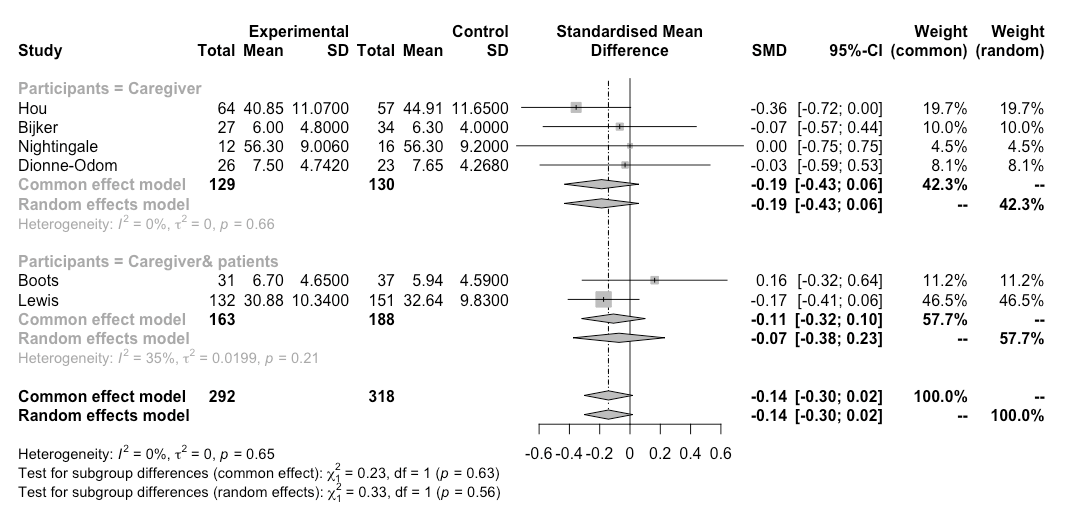


Subgroup 6- patients


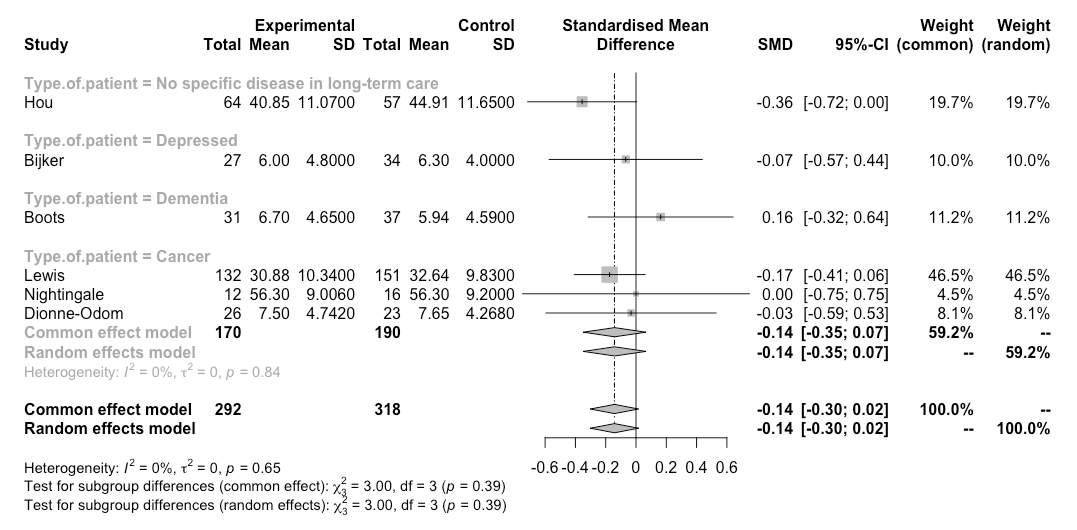


Subgroup 7- evaluation instruments


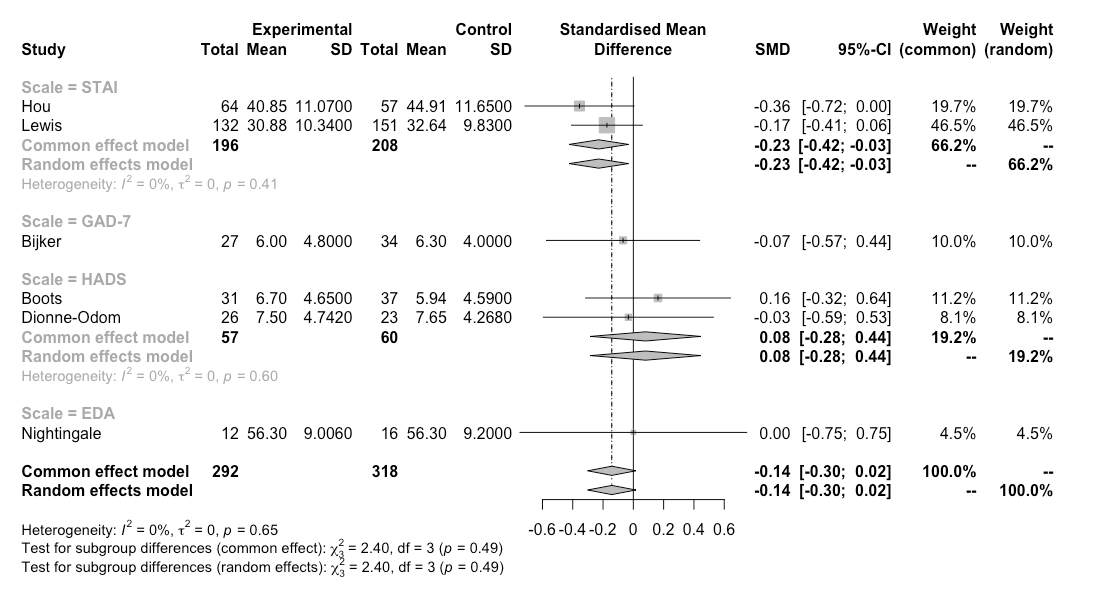


Subgroup 8- first follow-up time


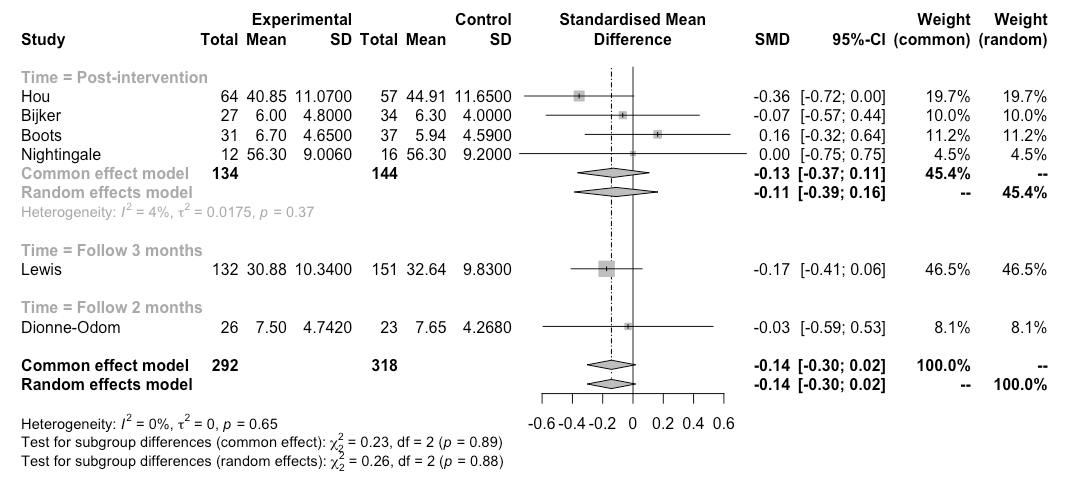

Supplement: Supplementary file 3 — Additional file 3. Meta-analysis for anxiety. [file 12877_2023_4614_MOESM3_ESM.docx]
