# Supplementary material for: Randomized controlled trials on promoting self-care behaviors among informal caregivers of older patients: a systematic review and meta-analysis
Source: BMC Geriatr. 2024 Jan 23;24:86. doi: 10.1186/s12877-023-04614-6 (PMC10804633; doi:10.1186/s12877-023-04614-6)

Additional file 4. Meta-analysis of depression

Forest plot


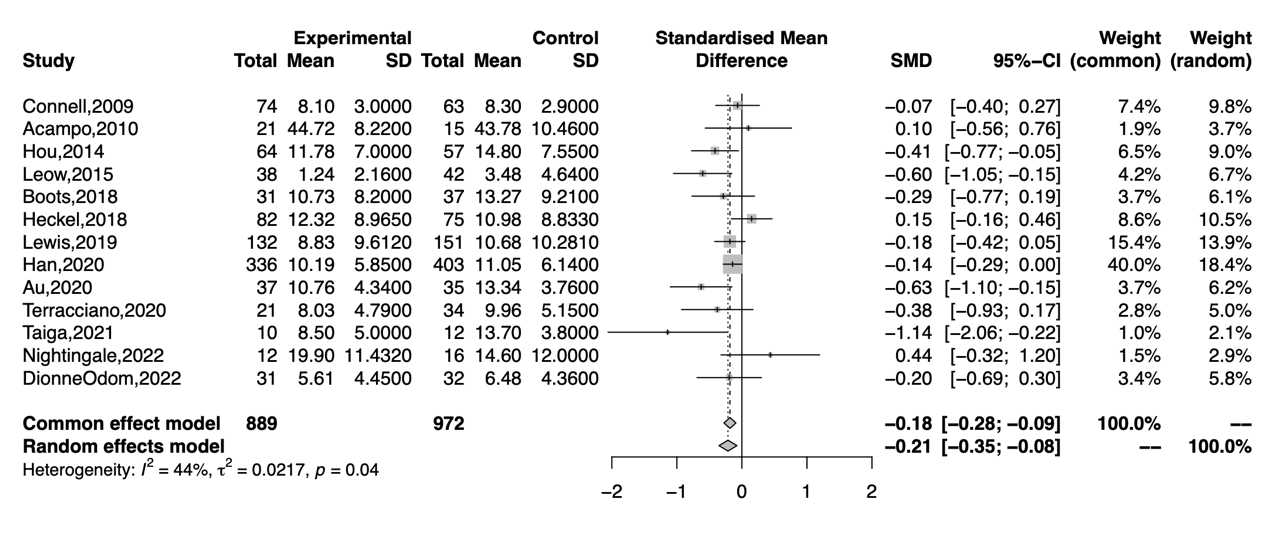


Funnel plot


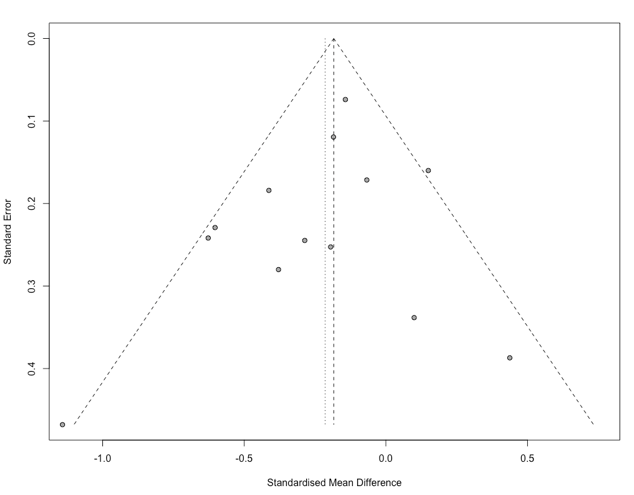


Sensitivity analysis (exclude low-quality studies)


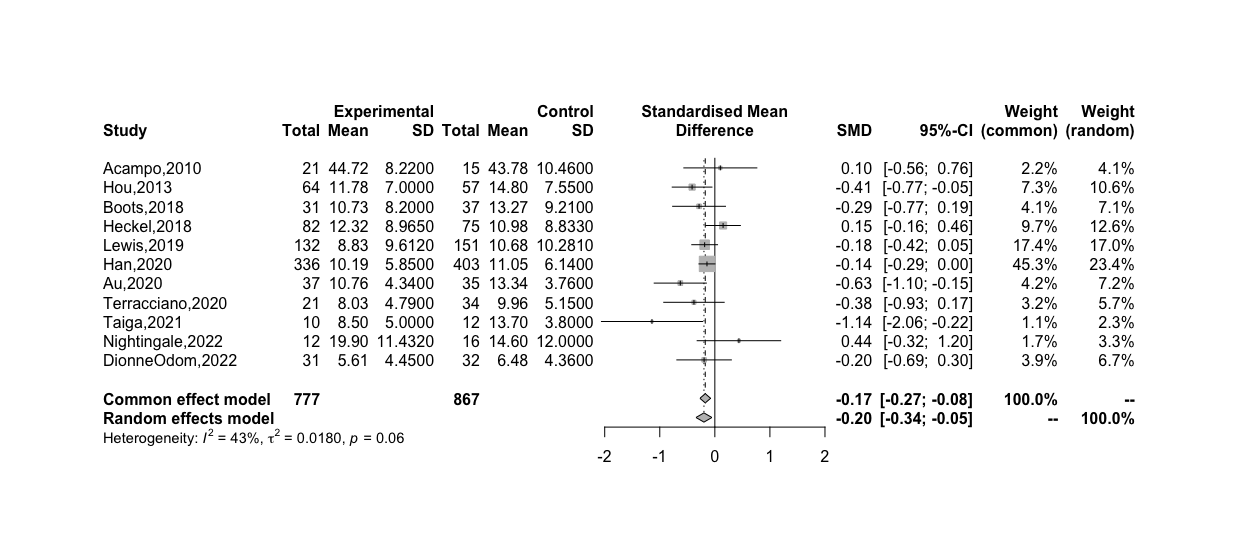


Subgroup analysis of depression

Table 1 Subgroup analysis of depression

|  | Coding | K (number) | SMD | 95% CI | Q (between groups) | P-value (between groups) |
| --- | --- | --- | --- | --- | --- | --- |
| Country | USA | 5 | -0.145 | [-0.311; 0.021] | 16.840 | 0.009*** |
|  | The Netherlands | 2 | -0.154 | [-0.542; 0.235] |  |  |
|  | Hong Kong, SAR, China | 2 | -0.492 | [-0.779; -0.205] |  |  |
|  | Singapore | 1 | -0.603 | [-1.052; -0.154] |  |  |
|  | Australia | 1 | 0.150 | [-0.164; 0.463] |  |  |
|  | Korean | 1 | -0.143 | [-0.288; 0.002] |  |  |
|  | Japan | 1 | -1.142 | [-2.060; -0.225] |  |  |
| Intervention | Online intervention | 4 | -0.114 | [-0.366; 0.137] | 1.000 | 0.607 |
| forms | Face-to-face intervention | 6 | -0.276 | [-0.586; 0.034] |  |  |
|  | Combination of online and face-to-face intervention | 3 | -0.284 | [-0.564; -0.005] |  |  |
| Intervention | Within one month or less | 3 | -0.502 | [-0.973; -0.032] | 5.890 | 0.053 |
| duration | One to three months | 8 | -0.222 | [-0.370; -0.075] |  |  |
|  | Over 3 months | 2 | 0.049 | [-0.181; 0.278] |  |  |
| Type of | Family caregiver | 10 | -0.277 | [-0.408; -0.146] | 9.020 | 0.003*** |
| caregiver | Informal caregiver | 3 | 0.177 | [-0.089; 0.443] |  |  |
| Participants | Caregiver | 7 | -0.178 | [-0.294; -0.062] | 0.150 | 0.700 |
|  | Caregiver& patients | 6 | -0.234 | [-0.493; 0.026] |  |  |
| Type of | Dementia | 5 | -0.380 | [-0.666; -0.094] | 2.570 | 0.463 |
| patients | Parkinson | 1 | 0.100 | [-0.563; 0.763] |  |  |
|  | Cancer | 5 | -0.119 | [-0.406; 0.167] |  |  |
|  | No specific disease in long-term care | 2 | -0.225 | [-0.469; 0.018] |  |  |
| Evaluation | CES-D | 10 | -0.196 | [-0.343; -0.050] | 3.800 | 0.284 |
| instruments | SDS | 1 | 0.100 | [-0.563; 0.763] |  |  |
|  | DASS | 1 | -0.603 | [-1.052; -0.154] |  |  |
|  | HADS | 1 | -0.195 | [-0.690; 0.300] |  |  |
| Measure | Post-intervention | 8 | -0.275 | [-0.467; -0.083] | 1.180 | 0.881 |
| time | Follow 1 month | 2 | -0.209 | [-0.946; 0.528] |  |  |
|  | Follow 2 months | 1 | -0.195 | [-0.690; 0.300] |  |  |
|  | Follow 3 months | 1 | -0.185 | [-0.419; 0.049] |  |  |
|  | Follow over 3 months | 1 | -0.067 | [-0.403; 0.269] |  |  |

P<0.1*, P<0.05**, P<0.01***

CES-D, Center for Epidemiologic Studies Depressive Scale; SDS, Self-rating Depression Scale; DASS, Depression Anxiety Stress Scales; HADS, Hospital Anxiety and Depression Scale (HADS).

Subgroup 1- country


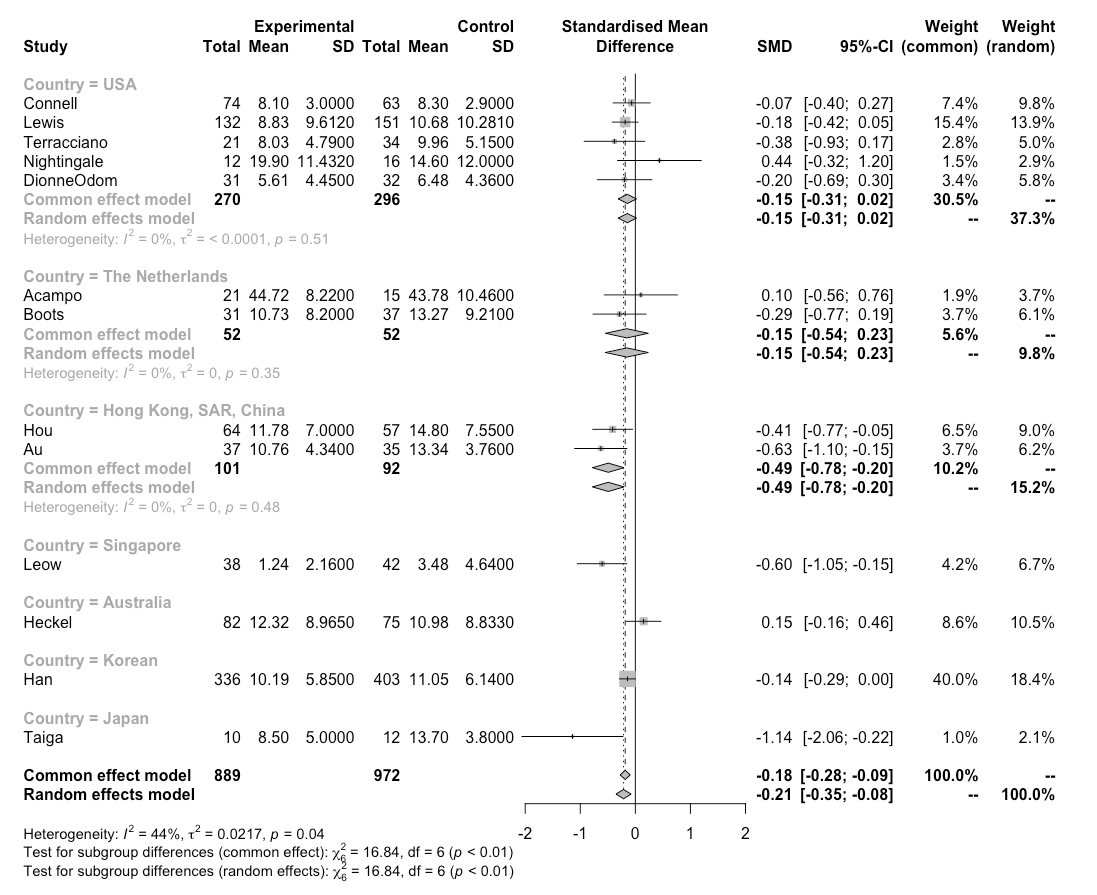


Subgroup 2 - intervention forms


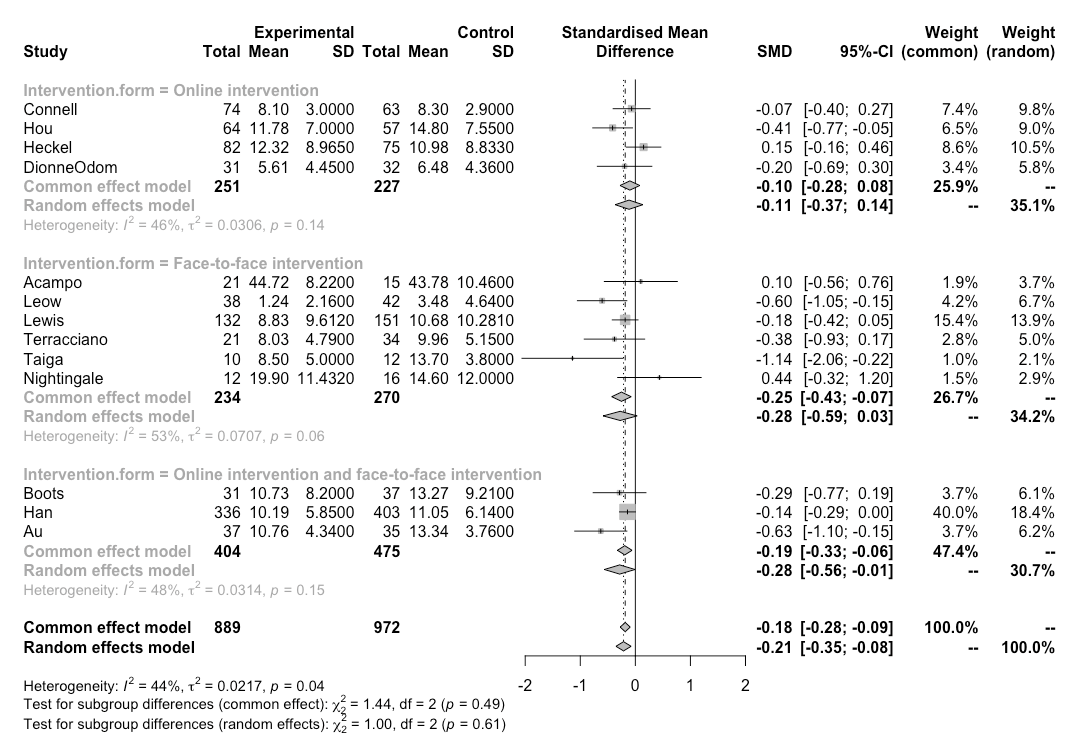


Subgroup 3- intervention duration


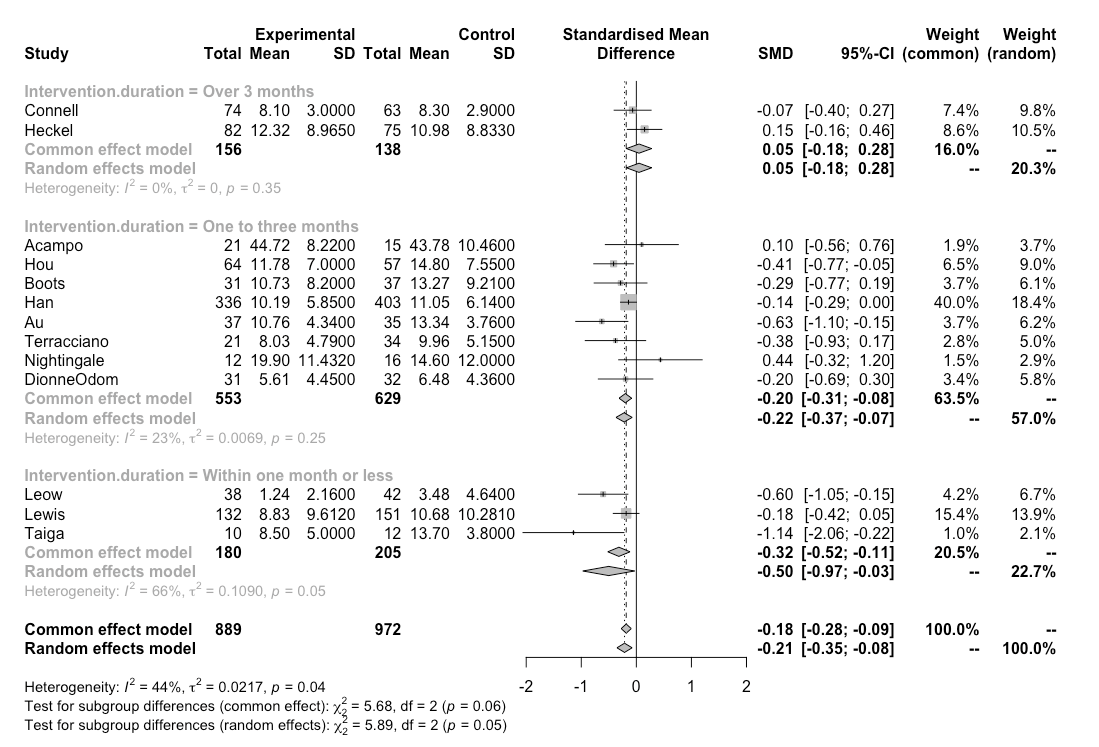


Subgroup 4- type of caregiver


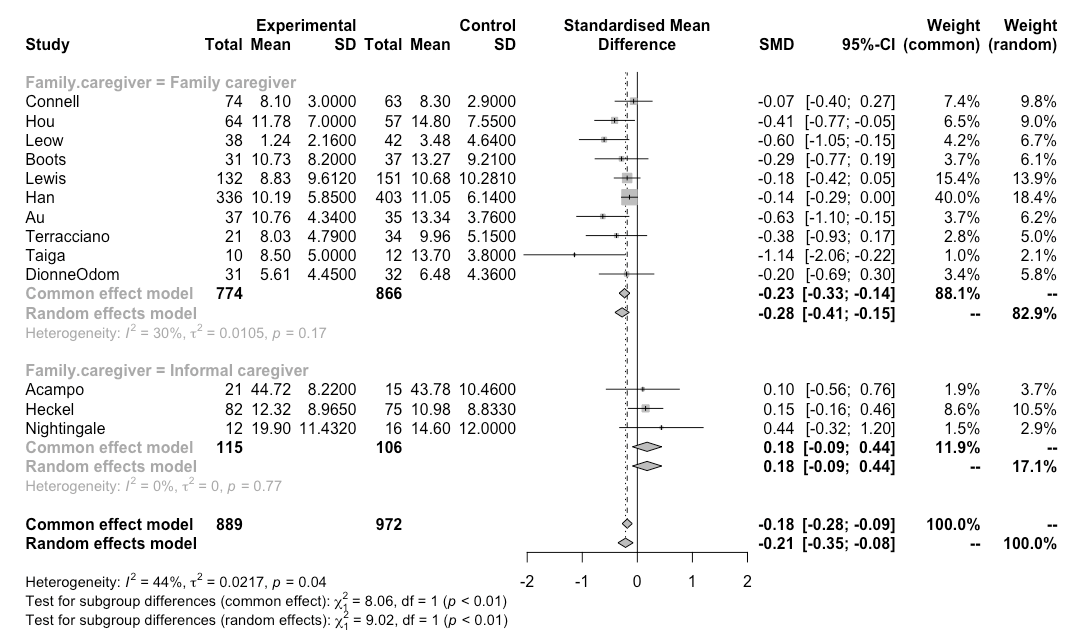


Subgroup 5- participants


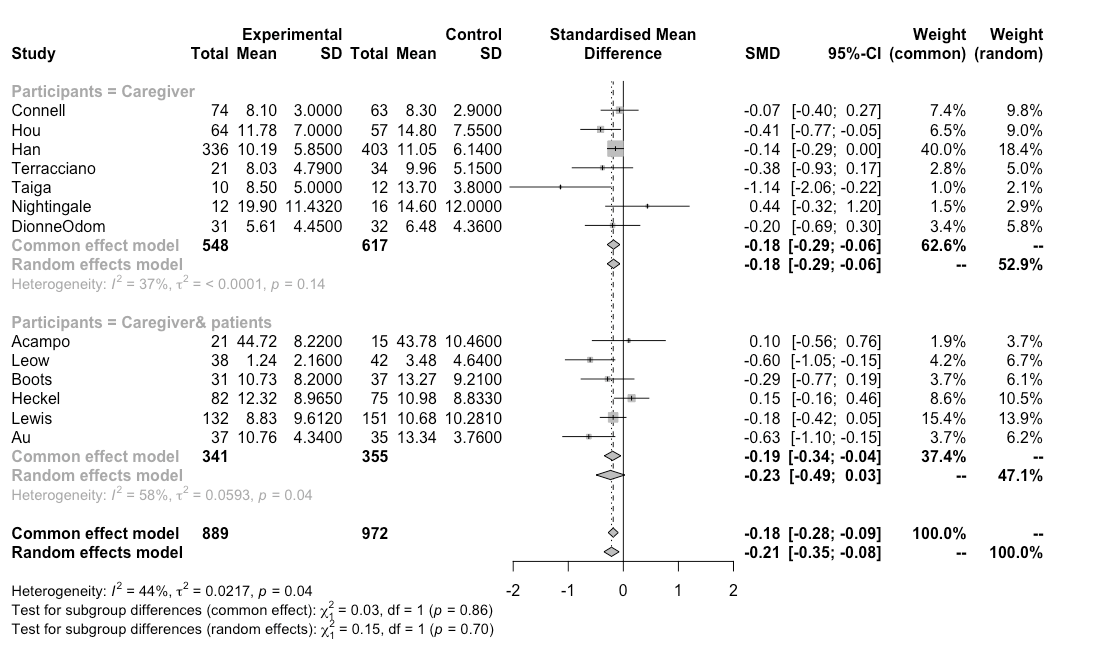


Subgroup 6- type of patients


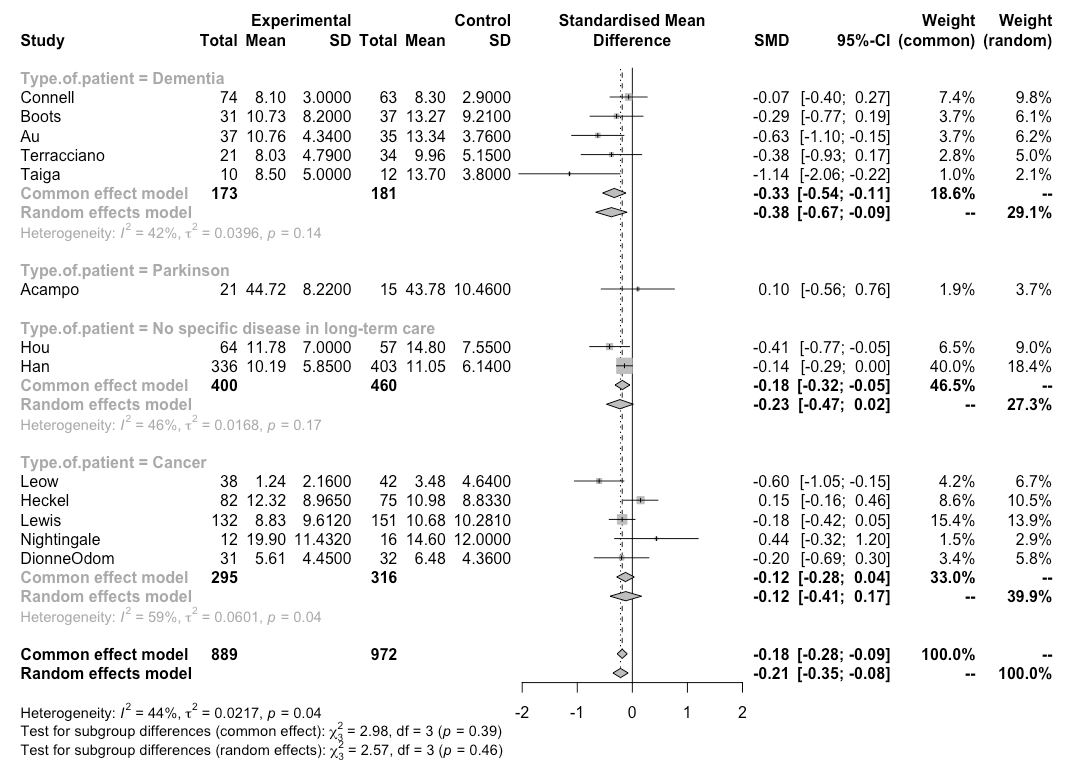


Subgroup 7- evaluation instruments


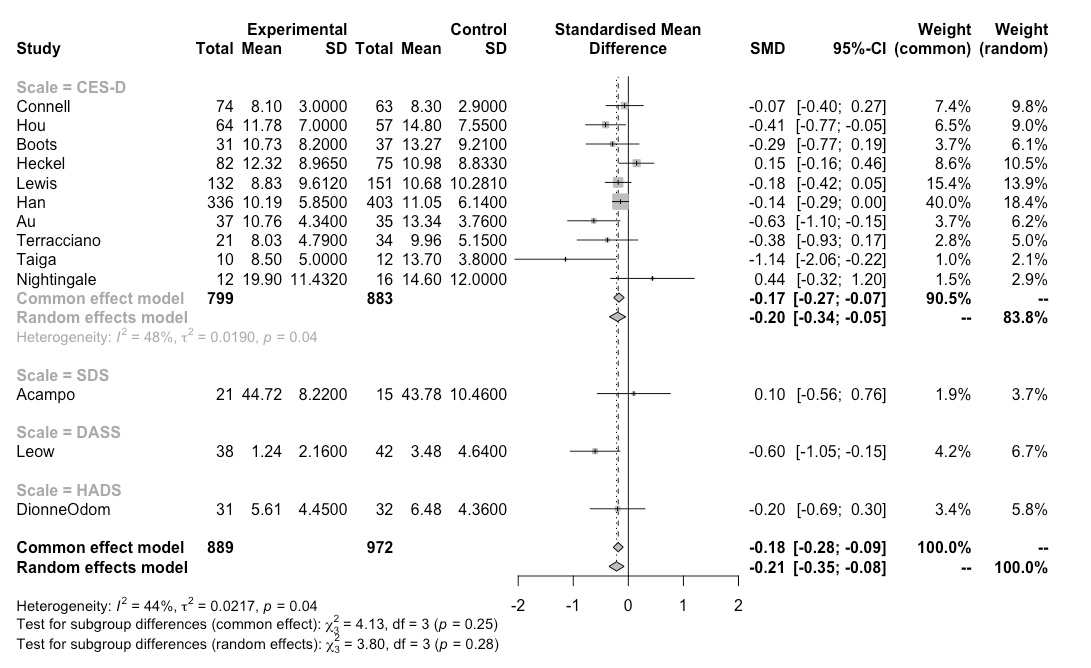


Subgroup 8- first follow-up time


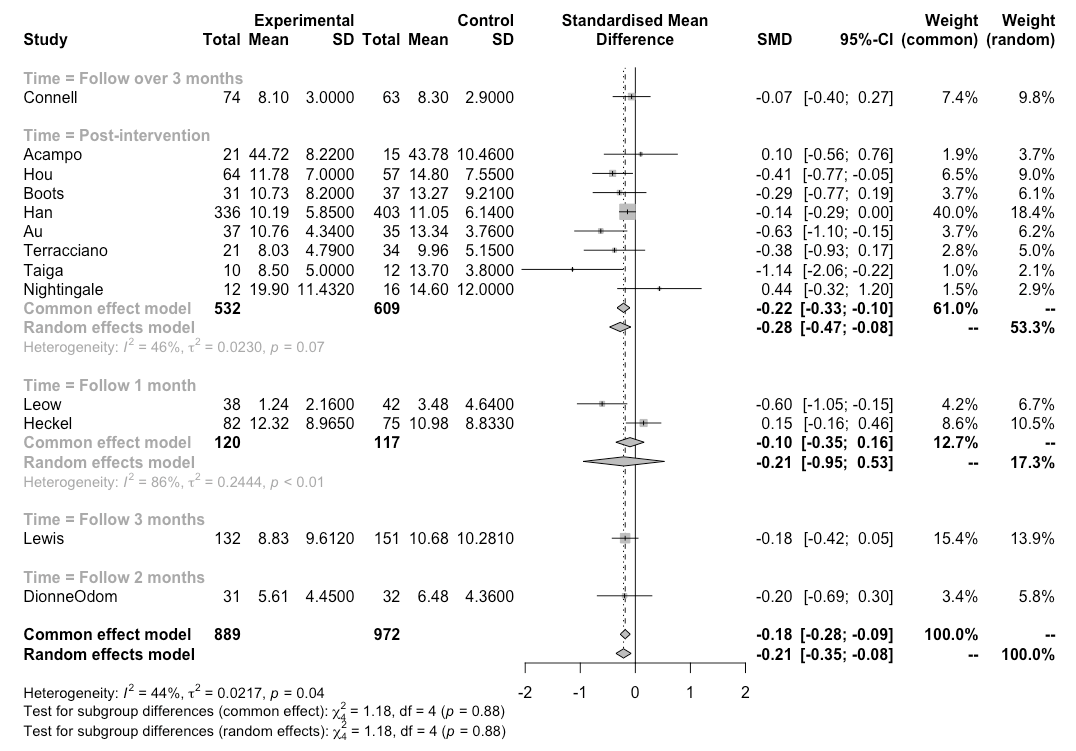

Supplement: Supplementary file 4 — Additional file 4. Meta-analysis of depression. [file 12877_2023_4614_MOESM4_ESM.docx]
